# Migration and family planning in the state with highest total fertility rate in India

**DOI:** 10.1186/s12889-020-09906-9

**Published:** 2020-11-30

**Authors:** Bidhubhusan Mahapatra, Niranjan Saggurti, Raman Mishra, Monika Walia, Saradiya Mukherjee

**Affiliations:** Population Council, B86, 2nd Floor, New Delhi, 110024 India

**Keywords:** Migration, Family planning, Contraceptive, Bihar

## Abstract

**Background:**

This study examined the relationship between male out-migration and family planning (FP) behaviour of women in rural Bihar.

**Methods:**

Data was collected from 937 currently married women aged 15–34 years from two districts of Bihar, namely Nawada and Gopalganj. Respondents were selected through a multi-stage systematic sampling and were recruited from both low and high male out-migration blocks. Differences in FP outcomes—use of modern contraceptive methods, intention to use contraceptives in next 12 months and access to FP services—were assessed by volume of migration, husband’s migration status, frequency of return, and duration of husband’s stay at home during visits.

**Results:**

Women with migrant husbands were about 50% less likely to use modern contraceptive methods. Further, the odds of using modern contraceptives was about half among women with migrant husbands if they resided in high out-migration areas (HMA) than low out-migration areas (LMA) (15% vs 29%, AOR: 0·50, *p* = 0·017). A higher proportion of women with migrant husbands, specifically from HMA, reported greater intention of using contraceptives in next 12 months than their counterparts (37% vs 23%, AOR: 1·83, *p* = 0·015). Similarly, access to FP services was negatively associated with the volume of male out-migration, specifically for women with migrant husbands.

**Conclusions:**

The migratory environment as well as the migration of husbands affect contraceptive use and access to FP services among women. Given that a significant proportion of married males leave their home states for work, it is imperative that FP programs in migration affected areas plan and implement migration-centric FP implementation strategies.

**Supplementary Information:**

The online version contains supplementary material available at 10.1186/s12889-020-09906-9.

## Background

India launched its family planning (FP) program in 1952 [1–3], starting with birth control programs and later expanding to include mother and child health, nutrition and family welfare. Since then, several modifications have been made to the program to minimize the growth rate of the ever-increasing population [1]. However, even after more than 60 years of the program, its impact remains non-uniform, and India is yet to achieve the replacement level fertility, and unmet need for contraception remain high [3, 4]. The Government of India in 2012, articulated its commitment to contribute towards an additional 48 million new users of contraceptive by 2020, comprising 40% of the global goal of 120 million [5]. To reach this goal, the required annual rate of increase in modern contraceptive prevalence rate (mCPR) in 2012 was estimated to be almost twice the current growth rate of 1% [5]. Concomitant to the national program, the state of Bihar also implemented the FP program under the guidance of Government of India. However, performance of the family planning program has been mediocre; mCPR reduced from 29% in 2005–06 to 23% in 2015–16 [6, 7]. In fact, Bihar has the highest fertility among Indian states with total fertility rate of 3·4 [8]. The situation in rural Bihar is worse than urban Bihar. While there have been ongoing discussions in and outside Bihar on what could have been done better in FP programs, it is important to look beyond the traditional factors considered for developing FP strategies. There needs to be more strategic rethinking to identify socio-cultural or demographic factors that need to be prioritized.

One of the factors that could have played a role in low mCPR is male out-migration. A great deal of evidence from Nepal, sharing an open border with Bihar, suggests that women with migrant husbands were less likely to use contraceptives and access FP services though they had greater autonomy than those with resident husbands [9–12]. Primary research conducted in Nepal also noted that women with migrant husbands were less likely to discuss FP issues with their spouses [10, 13, 14]. Other studies in Nepal documented that conversation on reproductive health and contraceptive use was dependent on frequency of the husband’s visit to the home state, and duration and destination of migration [12–14]. Likewise, there is evidence from other parts of the world examining the relationship between male migration, fertility and contraceptive use [15–17]. While evidence linking migration and FP is not available in India, researchers have documented the role of migration in transmission of HIV in Bihar and other parts of India [18–23]. Based on the premise of these empirical research studies and contextual similarity between Bihar and Nepal, this study hypothesized that male out-migration plays a role in FP behaviour of women in Bihar as well. In this backdrop, this study examined the macro and micro effects of male out-migration on contraceptive use behaviour and access to FP services among women in rural Bihar. The study aimed to answer the following questions: (i) are contraceptive use behaviour and access to FP services among women with migrant husbands different from those with resident husband? (ii) are there specific characteristics of migration associated with contraceptive use behaviour and access to FP services? and (iii) how does the migration environment affect contraceptive use behaviour and access to FP services?

## Methods

### Study setting

**Fig. 1:**
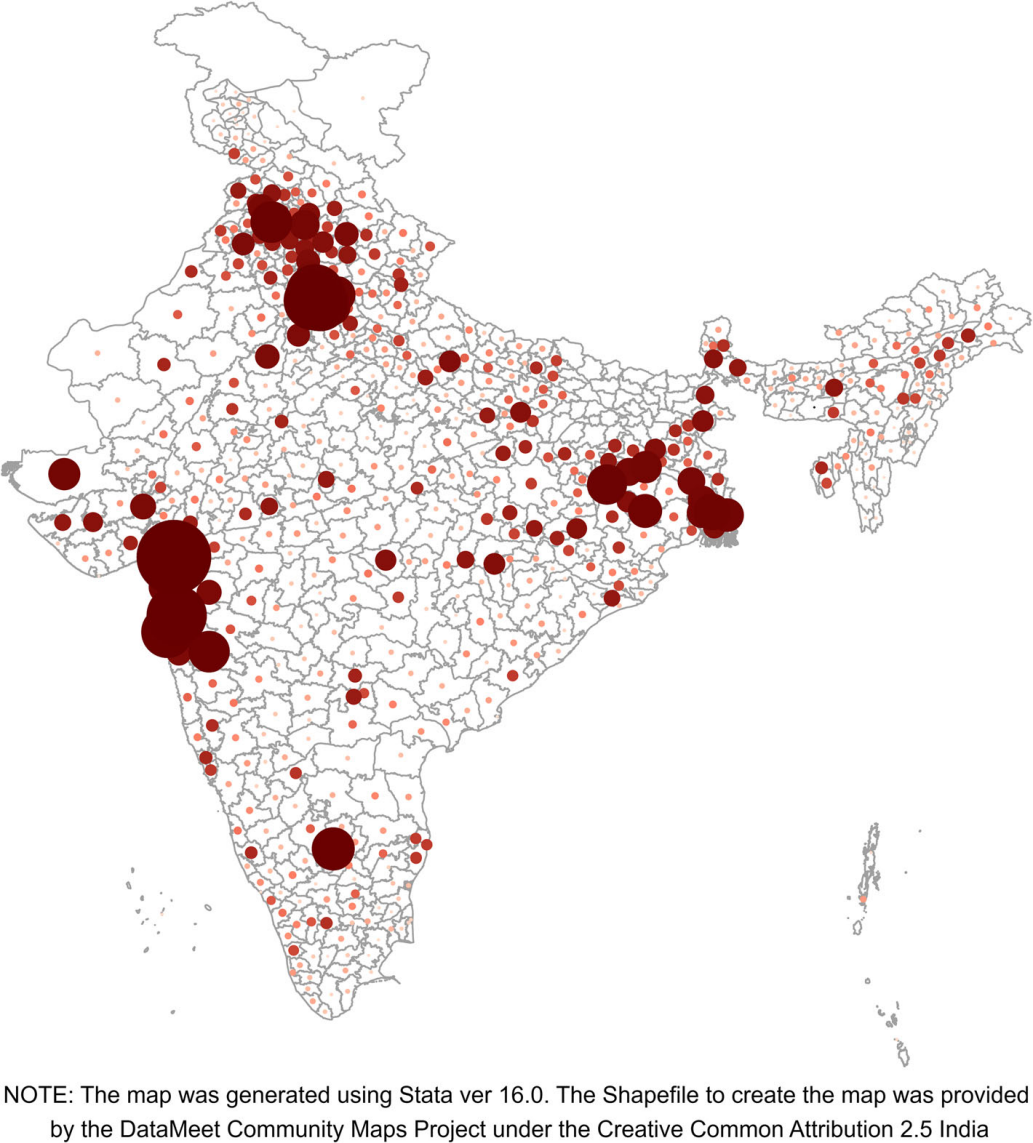
Volume of male out-migration from Bihar to other India districts, Census 2011 (Authors’ own creation)

The state of Bihar is located in the Eastern part of India and consists of 38 districts, with a population density of 1102/km^2^ [24]. Nearly 89% of the population resides in rural areas. The state has one of the highest migration rates in India [24, 25]. Migration from the state mostly takes place from rural areas to urban towns/cities across India [24, 25]; cities like Delhi and neighbouring towns, Mumbai and Thane in Maharashtra, and Surat in Gujarat are top three destinations for these migrants (Fig. 1). Migrants from Bihar stay away from home at their place of work for 13 years on an average, and majority of them return home once or twice in a year [26]. In terms of family planning, the state is amongst the poor performer states of India. Only 24% of women aged 15–49 years reported using a modern contraceptive, more than 85% of this was female sterilization [27]. About 21% women had unmet need for family planning [27]. Accredited Social Health Activists (ASHAs) are the key person at village level to reach out to women and deliver family planning services to women. The study was conducted in two high male out-migration districts of Bihar, namely Nawada (Southern Bihar) and Gopalganj (Northern Bihar). The male migration from these two districts is nearly the same: 36% from Nawada and 33% from Gopalganj. However, the use of modern contraceptives is quite different in these two districts: 29% in Nawada and 9% in Gopalganj [28, 29]. The socio-economic conditions in both districts were similar [28, 29]. For example, in both districts, three-fifths (57%) of the households had access to electricity, about one-quarter (26–28%) used improved sanitation facility, and one-fifth (20–21%) used clean fuel for cooking. A little more than a quarter of women in both districts had 10 or more years of schooling [28, 29].

### Study design

A formative research with qualitative study followed by quantitative survey was conducted. Qualitative research was conducted between April–July 2019, while the quantitative survey was conducted between June–July 2019. Both qualitative and quantitative data were collected from two high (Nawada: Sirdala and Warsaliganj; Gopalganj: Bhore and Phulwaria) and two low male out-migration blocks (Nawada: Hisua and Rajauli; Gopalganj: Kuchaikote and Thawe). These blocks were selected after consultation with the District Statistical Officer and District Labour Officer. The qualitative research included focus group discussions and key informant interviews with currently married women of migrant husbands, currently married men, community leaders, and FP service providers, to understand the issues around contraceptive use including FP service provisions in the context of migration. The findings of qualitative research helped in developing the tools for the quantitative survey.

A multi-stage sampling approach was used in the quantitative survey; in the first stage, six villages in each block were selected systematically using probability proportional to size approach. In the second stage, 20 households with eligible women i.e. currently married women aged 15–34 years were selected. If there was more than one currently married woman in a selected household, only one woman was selected for the interviewed. House-listing was conducted to identify households with eligible respondents. During house-listing, the head of the household was asked to provide name, age, and sex of all members, including current and last place of work for male members who were 15 years or above, and their marital status. Using the information on current and last place of work and current marital status, women in a household were categorised as having non-migrant, active migrant and returned migrant husbands. Households where more than one eligible woman was available, one was selected randomly. In order to have an appropriate sample size for analysis, the study selected 40% women with non-migrant husbands, 30% with active migrant husbands, and 30% with returned migrant husbands for the interview. Post-survey weights were computed to adjust for the population distribution by migration type in the sampled data. Structured tools were used to list household members and interviews with eligible women. The quantitative survey collected data on migration patterns, contraceptive use, method mix and access to FP services. Given the study had specific focus on migration and family planning, a specialized tool was developed (Supplement 1). The tool was field tested before implementing in the actual survey. During house-listing, 11,944 households were enumerated. The survey interviewed 937 women with a response rate of 98%.

### Measures

Three outcome measures are used in the study to understand the role of migration on FP behaviour: (a) currently using any modern contraceptive method, (b) intending to use a contraceptive in next 12 months, and (c) access to FP services.

Current use of modern contraceptive methods was assessed by asking all women whether they were currently using any contraceptive method, followed by type of methods being used. Women who reported using any modern method of contraception (male and female sterilization, injectables, intrauterine devices (IUDs/PPIUDs), contraceptive pills, implants, female and male condoms, standard days method, lactational amenorrhoea method, and emergency contraception) were considered as modern contraceptive users and coded as 1, else coded as 0.

Women who were not using any modern contraceptives were further asked if they intend to use a contraceptive in next 12 months. Those who responded affirmative was coded as 1 (intend to use contraceptive), else coded as 0.

A composite measure of access to FP services was created using four single item questions: (i) whether Front-Line Health Worker (FLW) talked about FP to women during in-person meeting in last 3 months, (ii) women reported easy to access modern spacing methods in terms of procurement cost, (iii) women reported easy to access modern spacing methods in terms of distance to place of availability and (iv) women had heard FP messages through mass media in past 6 months. These individual items were recoded into new items with response categories 0/1, with 1 representing access. Subsequently, they were added up to create a score of access to FP services. Women whose score was above two were considered to have access to FP services (coded as 1).

Migration was measured at both micro and macro level. Migration status of the husband was used for micro level measurement. The study asked all respondents where their husband was working, with the following response categories: working in the village, working outside the village but within the district, working outside the district but within the state, working outside state. If the husband was currently working outside the village, women were further asked if the husband was currently visiting his native place. If the husband was visiting home at the time of survey, women were asked if the husband planned to go back to his work location. Women whose husbands were working outside the state and planning to go back to their work location, if currently visiting, were considered to have a migrant husband, else considered to have resident husbands. The macro-level measurement included a measure of volume of migration at the village level. During the house-listing exercise, the study collected information on current place of work for all male members (aged 15+) of the household. Based on this information, villages above median male out-migration rate (30%) were considered as high male out-migration villages (HMA); else considered as one with low male out-migration (LMA).

In addition to the above two measures of migration, the study used three other variables of migration characteristics: frequency of husband’s return to native place, duration of stay when husband visited native place and current destination where husband was working. These three measures were computed only for women with migrant husbands. Women were asked where the husband was currently working, how frequently the husband returned home from the place of work and in general, how many days did the husband stay when he visited home. If the husband worked in a place that could be reached overnight or less time by train or bus journey, it was considered as working at a short distance destination; otherwise, considered as working long-distance. The response categories in the frequency of return variable was recoded into categories: frequent (husband returned multiple times a year) and infrequent (husband returned only once in a year). Similarly, the duration of stay data collected in days was recoded to < 30 and 30+ days.

The study also collected a range of socio-economic and demographic factors including age, education of women, caste, working status, number of living children, membership in self-help groups (SHGs), ownership of mobile phone, daily exposure to mass media and monthly household income. These variables were used and included as covariates while examining the effects of migration on FP outcomes.

### Statistical analyses

Bivariate and multivariate analyses were conducted. Bivariate analysis was used to present the profile of respondents by migration status of husband and prevalence of FP outcomes by migration characteristics of husband. A series of multiple logistic regression models were generated. For each combination of FP outcomes (use of modern contraceptives, intention to use and access to FP services) and migration characteristics (migration status of husband, frequency of return and duration of stay at home), separate logistic regression models were fitted. These models were fitted separately for LMA and HMA. Separate multilevel logistic regression models were fitted for each FP outcomes, with volume of migration as key predictor variable. These models were again run for each category of migration characteristics. All regression models were adjusted for age, education of women, caste, working status, number of living children, membership in SHGs, ownership of mobile phone, daily exposure to mass media and monthly household income. Results were presented in the form of percentages, adjusted odds ratios (AORs) and their corresponding 95% confidence interval (CI). All the analyses were carried out using STATA version 16·0 (StataCorp., College Station, TX, USA).

## Results

More than two-fifths (46%) of women had a migrant husband (Table 1). The profile of women did not differ much by their husband’s migration status. On average, women surveyed were 27 years old and had just over than two living children at the time of survey. A little more than one-third (36%) belonged to marginalized communities (Scheduled caste/tribe). About half (51%) of them had some level of formal education; a higher proportion of women with migrant husbands had formal education than those with resident husband (56% vs 48%, *p* = 0·03). Women with migrant husbands reported a higher monthly income than those with resident husbands (₹14,614 [US$209] vs ₹10,407 [US$149], *p* < 0·001).

**Table 1:** Profile of women by migration status of husband (*N* = 937)

Characteristics of women	Women with migrant husband (***N*** = 392)	Women with resident husband (***N*** = 545)	Overall
	% or Mean (SD)^**a**^	% or Mean (SD) ^**a**^	% or Mean (SD) ^**a**^
Age (in years)	26·8 (4·6)	26·4 (4·5)	26·6 (4·5)
Have formal education^§^	55·5	48·4	51·4
Belonging to Schedule Caste/Tribe	35·7	36·8	36·3
Currently working	9·4	11·6	10·7
Member of a self-help group	26·3	26·3	26·3
Owns a mobile phone^§^	81·8	64·7	71·8
Daily exposure to mass media (TV/Radio/Newspaper)	28·7	26·6	27·5
Monthly household income^§^	₹14,614 (8409)	₹10,407 (8334)	₹12,166 (8615)
Number of children	2·2 (1·4)	2·2 (1·5)	2·2 (1·4)
Husband visits native place frequently^b^	62·9	–	62·9
Husband stays for 30+ days during home visit^b^	48·4	–	48·4
Husband working at a long-distance destination	86·1	–	86·1

^a^*SD* Standard Deviation

^b^Computed only among women with migrant husband

^§^*p* < 0·05

The effects of the husband’s migration status and migration associated characteristics on women’s current use of modern contraceptive has been presented in Table 2. Women with migrant husbands were about 50% less likely to use modern contraceptive methods, regardless of whether they resides in high or low male out-migration area (LMA: 29% vs 34%, AOR = 0·51, *p* = 0·009; HMA: 15% vs 26%, AOR = 0·43, *p* = 0·002). The odds of using modern contraceptives was lower among women residing in HMA than in LMA, irrespective of whether they had a migrant or resident husband. For example, the odds of using modern contraceptives was 50% less among women with migrant husbands if they stayed in HMA than LMA (15% vs 29%, AOR: 0·50, *p* = 0·017). Similarly, women with migrant husbands in HMA were less likely to use modern contraceptives, irrespective of their husband’s frequency of return and stay during home visits.

**Table 2:** Effects of migration on modern contraceptive use among women by volume of migration

Characteristics	Low male out-migration area (LMA)		High male out-migration area (HMA)		HMA vs LMA
	%(N)	AOR (95% CI), ***p***-value^**€**^	%(N)	AOR (95% CI), ***p***-value^**€**^	AOR (95% CI), ***p***-value^**€**^
**Overall**	32·0 (446)		21·2 (491)		**0·58 (0·35–0·95),** ***p*** **= 0·030**
**Migration status of husband**					
Resident	34·3 (266)	Referent	26·0 (280)	Referent	**0·57 (0·36–0·91),** ***p*** **= 0·020**
Migrant	28·7 (180)	**0·51 (0·31–0·85),** ***p*** **= 0·009**	14·7 (211)	**0·43 (0·26–0·73),** ***p*** **= 0·002**	0·43 (0·15–1·27), *p* = 0·128
**Frequency of husband’s home visit**^**a**^					
Infrequent (Once or less in a year)	30·3 (62)	Referent	13·6 (84)	Referent	**0·27 (0·08–0·91),** ***p*** **= 0·034**
Frequent (Multiple visits within a year)	27·8 (118)	0·57 (0·24–1·36), *p* = 0·203	15·4 (127)	1·65 (0·62–4·43), *p* = 0·317	0·64 (0·31–1·31), *p* = 0·222
**Number of days husband stays during home visits**^**a**^					
< 30 days	27·0 (105)	0·76 (0·34–1·67), *p* = 0·491	10·0 (97)	0·52 (0·20–1·37), *p* = 0·187	**0·38 (0·16–0·94),** ***p*** **= 0·036**
30+ days	31·0 (75)	Referent	18·7 (114)	Referent	0·50 (0·21–1·21), *p* = 0·125
**Destination where husband has been working**^**a**^					
Short distance destination	30·6 (36)	Referent	33·2 (19)	Referent	1·62 (0·09–30·1), *p* = 0·748
Long distance destination	28·2 (145)	1·52 (0·56–4·16), *p* = 0·413	12·9 (192)	0·46 (0·14–1·52), *p* = 0·201	0·41 (0·13–1·28), *p* = 0·126

^**a**^Computed only among women with migrant husbands

^**€**^*AOR* Adjusted Odds Ratio, *CI* Confidence interval; AORs and CIs were estimated from multiple logistic regression models adjusted for age, education, caste, working status, membership in self-help group, ownership of phone, daily mass media exposure, monthly household income and number of children

The effects of the husband’s migration status and migration associated characteristics on women’s intention to use contraceptive methods in next 12 months has been presented in Table 3. A higher proportion of women with migrant husbands, specifically from HMA, reported intention of using contraceptives in next 12 months than their counterparts (37% vs 23%, AOR: 1·83, *p* = 0·015). Among women with migrant husbands, the intention to use was positively associated with frequent home visits of the husband. In HMA, the odds of intention to use were twice higher among women whose husbands stayed for less than 30 days than whose husbands stayed for longer periods (45% vs 28%, AOR: 2·14, *P* = 0·025).

**Table 3:** Effects of migration on intention to family planning use among women currently not using contraceptives by volume of migration

Characteristics	Low male out-migration area (LMA)		High male out-migration area (HMA)		HMA vs LMA
	%(N)	AOR (95% CI), ***p***-value^**€**^	%(N)	AOR (95% CI), ***p***-value^**€**^	AOR (95% CI), ***p***-value^**€**^
**Overall**	23·9 (446)		27·3 (491)		1·23 (0·81–1·87), *p* = 0·330
**Migration status of husband**					
Resident	26·6 (175)	Referent	23·4 (207)	Referent	1·04 (0·67–1·61), *p* = 0·878
Migrant	31·8 (129)	1·46 (0·84–2·54), *p* = 0·178	36·6 (180)	**1·83 (1·12–2·99),** ***p*** **= 0·015**	1·25 (0·75–2·08), *p* = 0·387
**Frequency of husband’s home visit**^**a**^					
Infrequent (Once or less in a year)	25·3 (43)	Referent	31·7 (72)	Referent	3·51 (0·32–38·9), *p* = 0·306
Frequent (Multiple visits within a year)	35·1 (86)	1·62 (0·58–4·56), *p* = 0·357	39·9 (108)	1·19 (0·60–2·35), *p* = 0·617	1·81 (0·70–4·70), *p* = 0·222
**Number of days husband stays during home visits**^**a**^					
< 30 days	30·4 (77)	1·29 (0·52–3·18), *p* = 0·581	45·4 (87)	**2·14 (1·10–4·14),** ***p*** **= 0·025**	**4·49 (1·05–19·3),** ***p*** **= 0·043**
30+ days	33·9 (52)	Referent	28·4 (93)	Referent	1·03 (0·21–4·98), *p* = 0·973
**Destination where husband is currently working**^**a**^					
Short distance destination	30·8 (25)	Referent	39·3 (13)	Referent	0·76 (0·11–5·15), *p* = 0·774
Long distance destination	32·0 (104)	1·19 (0·36–3·97), *p* = 0·771	36·4 (167)	0·88 (0·23–3·33), *p* = 0·854	1·23 (0·70–2·16), *p* = 0·475

^**a**^Computed only among women with migrant husbands

^**€**^*AOR* Adjusted Odds Ratio, *CI* Confidence interval; AORs and CIs were estimated from multiple logistic regression models adjusted for age, education, caste, working status, membership in self-help group, ownership of phone, daily mass media exposure, monthly household income and number of children

A little more than one-fifths (21%) of women had access to FP services (Table 4). Access to FP services was negatively associated with the volume of male out-migration, specifically for women with migrant husbands. In HMA, the odds of access to FP services was nearly half among women with migrant husbands than those with resident husbands (17% vs 23%, AOR: 0·46, *p* = 0·001). Further, women whose husbands returned infrequently, the likelihood of accessing FP services reduced by 90% if the women resided in HMA than in LMA (13% vs 27%, AOR: 0·10, *p* = 0·028). Similarly, among women whose migrant husbands stayed for 30 or more days while visiting home, the probability of accessing FP services reduced by 88% for those staying in HMA than in LMA (13% vs 21%, AOR: 0·12, *p* = 0·023).

**Table 4:** Effects of migration on access to family planning services among women by volume of migration

Characteristics	Low male out-migration area (LMA)		High male out-migration area (HMA)		HMA vs LMA
	%(N)	AOR (95% CI), ***p***-value^**€**^	%(N)	AOR (95% CI), ***p***-value^**€**^	AOR (95% CI), ***p***-value^**€**^
**Overall**	22·4 (446)		20·3 (491)		0·65 (0·40–1·04), *p* = 0·073
**Migration status of husband**					
Resident	19·2 (266)	Referent	23·0 (280)	Referent	0·95 (0·53–1·70), *p* = 0·860
Migrant	27·1 (180)	1·29 (0·77–2·16), *p* = 0·333	17·0 (211)	**0·46 (0·29–0·72),** ***p*** **= 0·001**	**0·31 (0·14–0·71),** ***p*** **= 0·005**
**Frequency of husband’s home visit**^**a**^					
Infrequent (Once or less in a year)	27·1 (62)	Referent	13·2 (84)	Referent	**0·10 (0·01–0·78),** ***p*** **= 0·028**
Frequent (Multiple visits within a year)	27·0 (118)	0·67 (0·28–1·61), *p* = 0·369	19·4 (127)	1·58 (0·63–3·94), *p* = 0·331	**0·32 (0·11–0·92),** ***p*** **= 0·034**
**Number of days husband stays during home visits**^**a**^					
< 30 days	31·6 (105)	1·31 (0·58–2·97), *p* = 0·512	22·2 (97)	**3·76 (1·45–9·76),** ***p*** **= 0·006**	0·24 (0·06–1·02), *p* = 0·054
30+ days	20·8 (75)	Referent	12·6 (114)	Referent	**0·12 (0·02–0·74),** ***p*** **= 0·023**
**Destination where husband is currently working**^**a**^					
Short distance destination	17·9 (36)	Referent	3·3 (19)	Referent	0·32 (0·01–10·6), *p* = 0·522
Long distance destination	29·3 (145)	2·62 (0·76–9·05), *p* = 0·129	18·3 (192)	3·79 (0·24–60·8), *p* = 0·346	**0·28 (0·10–0·78),** ***p*** **= 0·015**

^**a**^Computed only among women with migrant husbands

^**€**^*AOR* Adjusted Odds Ratio, *CI* Confidence interval; AORs and CIs were estimated from multiple logistic regression models adjusted for age, education, caste, working status, membership in self-help group, ownership of phone, daily mass media exposure, monthly household income and number of children

## Discussion

The state of Bihar has been grappling with several health and developmental issues. While FP programs in Bihar is being implemented for decades, the decline in mCPR in recent years has caused concerns among policy makers and program implementers [30]. This study brings a new perspective to the FP program in Bihar by examining effects of migration on FP outcomes. The study found that the migration environment is an important determinant of FP outcomes in Bihar. It showed that not only migration of husband, but also the environment where a woman resided influenced her FP behaviour. This is clearly reflected in the fact that women in HMA were significantly less likely to use modern contraceptives than those in LMA, regardless of their husband’s migration status. Further, the contraceptive use was similar among women with resident husbands in LMA and those with migrant husbands in HMA. The effects of the migration environment were also seen on the access to FP services; where particularly women with migrant husbands and residing in HMA were more likely not to have access to FP services than their counterparts. This reflected the existence of a systematic bias in providing FP services. This could be because FLWs may not be perceiving the importance of outreach as most husbands are away from the village. Prior studies have documented that the outreach by FLWs on FP is very poor in Bihar [7] and such systematic patterns of outreach can further worsen the situation of FP in Bihar.

The study found that women with migrant husbands were more inclined to use contraceptives than women with resident husbands. This was in line with the research conducted in Nepal that also showed a negative association between contraceptive use and the husband’s migration status [10–12]. In contrast to the Nepal study by Shattuck et al. [10], this study indicated that women with migrant husbands had higher intention of using contraceptives than their counterparts. One may argue regarding the necessity of using contraceptives when the husband was away. Undoubtedly in the absence of the husband, women may feel internally stigmatised to access FP methods due to existing social contexts in rural communities. The internal stigma may actually influence the intention of women to discuss with FLWs on family planning. During the qualitative phase of the study, several women having migrant husband reported about consequences if their family members get to know about women’s attempt to access family planning method when husband is away. On the other hand, this study points towards the prolonged non-use of contraceptives owing to the husband’s absence and how it diminishes contact with FLWs, which can lead to under-preparedness to procure FP methods when the husband arrives. Post-hoc analysis suggests that women get to know about their husband’s return about 30 days in advance. And even when husband is away the communication around FP is generally missing [14]. Further, only 13% of women met FLW to discuss FP methods in 3 months prior to study. Given the poor access to FP services, particularly by women with migrant husbands in HMA, meeting an FLW in those 30 days looks like a distant possibility. This means even the one-third of women with migrant husbands and who intend to use a method in the next 12 months, may not actually end up using when their husbands arrive.

The analysis also reflected the specific migration characteristics that are linked to various FP outcomes. For example, the number of days the husband stayed during the home visit as a key predictor for intention to use and access to FP services. In HMA, fewer days (< 30 days) stayed at home by husbands during their visits was positively associated with access to FP services and higher intention to use. However, this had no effect on contraceptive use. Post-hoc analysis showed that a higher proportion of women whose husbands stayed for a month or less were working in a nearby state, as compared to those staying at home for longer durations (22% vs 12%). Therefore, women whose husbands stayed for < 30 days could visit home more frequently. One can safely assume that such frequent visits could have resulted in frequent sexual encounters which may have propelled women to reach out to ASHA workers for seeking advice on FP services. Though this reflected their intention to use, the actual use of contraceptives was missing. Surprisingly, the place of destination had no association with any FP outcomes, possibly due to low cell frequency of short-distance migrants. Prior researches have documented that when husbands are long-distance migrants, the communication between couples on FP issues takes a backseat and hence, has a negative effect on contraceptive use [12, 14, 31]. While the analysis suggests a relationship between the destination and current contraceptive use to be in that direction, this relationship was not statistically significant. It is suggested that future research should collect more data to specifically test this relationship to test the hypothesis.

Migration in Bihar, much like other parts of India, is continuously changing in size, form and place, due to growing urbanization and lack of livelihood opportunities in rural areas supplemented by climate change. While it is the primary responsibility of FLWs to reach out to women irrespective of their husband’s migration status, in Bihar, it seems like ASHAs don’t prioritize reaching out to women in HMA and specifically those having a migrant husband. Therefore,, it is imperative for FP programs in Bihar and other migration affected areas to prepare migration-centric implementation strategies. Though FP programs in Bihar are constantly evolving, there needs to be paradigm shift in the approach and rethinking of implementation. A starting point in this can be mapping out villages with high density of migrant households. The mapping should collect information on destination, frequency and month of return besides other basic demographic details. This would help devising appropriate outreach strategies. The FLWs’ outreach to women, particularly in migration affected areas, should be re-strategized with emphasis on contacting women whose husbands are expected to return soon. Given that it is husbands who refuse to use contraceptives, there should be provisions of emergency contraceptives at the village level so that women can still prevent getting pregnant if they have unprotected sex. Finally, the attitude towards acceptance of family planning among couple is important. Therefore, it is important to understand the reasons for non-use of contraceptives. While empirical evidence highlights husband’s refusal to use contraceptive, infrequent sex, and concerns around side effects as key reasons behind non-use of contraceptives [7], they need to be re-investigated in the context of migration.

## Conclusion

This study contributes to the existing body of literature which has documented the relationship between migration and health. The study demonstrated how areas with high volume of male migration are at a disadvantage in accessing FP services, which leads to poor contraceptive use among women. Ongoing and future FP programs in Bihar need to consider this new perspective and devise strategies that address the FP needs of women in areas affected by migration.

## Supplementary Information

**Additional file 1.**

## Data Availability

The datasets analysed during the current study are not publicly available as the team is working on other research papers but are available from the corresponding author on reasonable request.

## Abbreviation

AOR: Adjusted Odds Ratio
ASHA: Accredited Social Health Activist
CI: Confidence Interval
FLW: Front-line Health Worker
FP: Family Planning
HIV: Human Immunodeficiency Viruses
HMA: High male out-migration villages
IUD: Intra-Uterine Devices
LMA: Low male out-migration villages
mCPR: Modern Contraceptive Prevalence Rate
PPIUD: Post-Partum Intra-Uterine Devices
SHG: Self-Help Group
