# Supplementary material for: Migration and family planning in the state with highest total fertility rate in India
Source: BMC Public Health. 2020 Nov 30;20:1826. doi: 10.1186/s12889-020-09906-9 (PMC7708902; doi:10.1186/s12889-020-09906-9)
Supplement: Supplementary file 1 — Additional file 1. [file 12889_2020_9906_MOESM1_ESM.pdf]

Identifying strategies for effective family planning program in out-migration areas in Bihar
Questionnaire for currently married women aged 15-34 years

Block 0: Identification

#	Questions	Response		Codes	Skip
B1	Household ID	ID________________			
B2	Woman's ID	ID________________			
B3	Name and code of the district				
B4	Name and code of block				
B5	Name and code of village/city				
B6	Name and code of the PHC				
B7	Name of the HH head	Name			
B8	Name of the respondent	Name			
B9	Date of interview (DD/MM/YYYY)	Day	Month	Year	
B10	Place of residence	Rural<br>Urban		1<br>2	
B	Interviewer's name and code	Name ____________________			

Block-1: Household and Respondent Characteristics

#	Question	Answers	Codes	Skip
101	How old are you?	Age in completed years		
102	Can you read and/or write?	Yes, can read only,<br>Yes, can write only,<br>Yes, can both read and write<br>No	1<br>2<br>3<br>4	
103	What is the highest standard/class you completed?	If less than 11, actual class<br>Higher secondary<br>Graduate<br>Diploma/ ITI/BTech or any other vocational studies<br>Masters/Post-graduate/Doctorate<br>Non-formal education	__<br>11<br>12<br>13<br><br>14<br>96	
104	Apart from household work do you do any work for which you get paid?	Yes<br>No	1<br>0	 ►**108**
105	What work do you do?	Casual labor in agriculture<br>Casual labor in Non-agriculture<br>Regular Salary/wage<br>Self-employed in agriculture<br>Self-employed in non-agriculture<br>Other (specify)________	1<br>2<br>3<br>4<br>5<br>7	
106	Do you usually do this throughout the year, seasonally or as and when needed?	Throughout the year<br>Part of the year/Seasonally<br>As and when needed	1<br>2<br>3	
107	How much do you earn from this work in a month?	Amount	____	
108	How many members are there in this household who usually live here?	Total number of members	____	
109	How many members are there in this household who usually stays in a different village/town/city?	Total number of members	____	
110	Beside you, are there any other women who is currently married and aged 15-49? If yes, how many such women are there in this household?	Number<br>None	__<br>0	

#	Question	Answers	Codes	Skip
111	Of these, how many women's husbands are staying outside the district?	Number of women	___	
112	What is the main construction material of the house?<br>**INSTRUCTION: RECORD OBSERVATION**	Grass/Leaves/Reeds/sod<br>Palm/Bamboo/Thatch<br>Uncovered adobe<br>Plastic/Fabric<br>Metal/CGI/Asbestos sheets<br>Wood/Wood planks/Plywood<br>Stone/bricks with mud<br>Straw and mud<br>Mud<br>Concrete/cement<br>Other (Specify) ___	11<br>12<br>13<br>14<br>15<br>16<br>17<br>18<br>19<br>20<br>97	
113	What is your religion**?**	Hindu<br>Muslim<br>Christian<br>Buddist<br>Others (Specify)	1<br>2<br>3<br>4<br>7	
114	What is your caste or tribe?	Caste/Tribe_______________<br>No caste/tribe<br>Don't know	1<br>2<br>8	 **►116**
115	Do you belong to a scheduled caste, scheduled tribe or OBC or none of these?	Scheduled caste<br>Scheduled tribe<br>Other back ward class<br>Other	1<br>2<br>3<br>7	
116	Do you own a mobile phone?	Yes<br>No	1<br>0	**►118**
117	Does anyone else in the household owns a mobile phone?	Yes<br>No	1<br>0	
118	What is the main source of your household income?	Agriculture/Horticulture<br>Remittance<br>Petty business<br>Daily wage laborer<br>Poultry<br>Livestock<br>Contractual work<br>Carpentry/Plumbing<br>Private job<br>Government job<br>Other salaried job<br>Others (Specify)	11<br>12<br>13<br>14<br>15<br>16<br>17<br>18<br>19<br>20<br>21<br>97	
119	How much is your household's monthly income from all the sources?	Amount	—	
120	Are you able to save money after covering all your expenses in a month?	Yes<br>No	1<br>0	 ►123
121	How much are you able to save in a month?	Amount	_____	
122	Where do you primarily save your money?	Bank<br>SHG<br>At house only<br>Cooperative<br>Post office<br>Other (Specify)	1<br>2<br>3<br>4<br>5<br>7	

#	Question	Answers	Codes	Skip
123	What is the **main source** of drinking water for members of your household?	**Piped water**<br>Piped into dwelling<br>Piped into compound, yard/plot<br>Public tap/standpipe<br>Tube well or borehole<br><br>**Dug well**<br>Protected well<br>Unprotected well<br><br>**Water from spring**<br>Protected spring<br>Unprotected spring<br><br>Rainwater collection<br>Tanker truck<br>Surface water e.g. river, pond, stream,<br>Bottled water<br>Other (Specify) _______	 11<br>12<br>13<br>14<br><br><br>15<br>16<br><br><br>17<br>18<br><br>19<br>20<br>21<br>22<br>97	
124	What kind of toilet facility do members of your household usually use?	**Flush or pour flush toilet**<br>Flush to piped sewer system<br>Flush to septic tank<br>Flush to pit latrine<br>Flush to somewhere else<br><br>**Pit latrine**<br>Pit ventilated improved bio-gas latrine<br>Pit latrine with slabs<br>Pit latrine without slab/open pit<br><br>Twin pit/composting toilet<br>No facility/ uses open space or field<br>Other (Specify) __________________	 11<br>12<br>13<br>14<br><br><br>15<br>16<br>17<br><br>18<br>19<br>97	 <br><br><br><br><br><br><br><br><br><br><br>►126
125	Do you share the toilet facility with other households?	Yes<br>No	1<br>0	
126	What type of fuel does your household mainly use for cooking?<br>?	No food cooked in the household<br>Electricity<br>LPG/Natural gas<br>Biogas<br>Kerosene<br>Coal/lignite<br>Charcoal<br>Wood<br>Straw/Shrubs/Grass<br>Agricultural crop waste<br>Dung cakes/Animal dung,<br>Others (Specify) ______	10<br>11<br>12<br>13<br>14<br>15<br>16<br>17<br>18<br>19<br>20<br>97	►129<br>►127<br>►127<br><br>►127<br>►127<br>►127<br>►127<br>►127<br>►127<br>►127<br>►127
126a	Has your family got the LPG connection under Ujjawala scheme?	Yes<br>No	1<br>0	
127	Where is your kitchen located?	Inside the house<br>In a separate building<br>Outside the house (open)	1<br>2<br>3	 ►128a<br>►128a
128	Does your household have a separate room that is used as kitchen?	Yes<br>No	1<br>0	
128a	Does your kitchen have ventilation or exhaust fan?	Yes<br>No	1<br>0	

#	Question	Answers	Codes	Skip
129	Does any member of this household own any agricultural land? If yes, are you doing farming on it or is it leased to someone else?, ?	Yes, but leased to someone else,<br>Yes, doing farming,<br>Yes, left barren,<br>No	1<br>2<br>3<br>0	
130	Are you a member of any Self-Help Group (SHG)?	Yes<br>No	1<br>2	 ►133
131	Since how many months you have been a member of SHG?	Months_____		
132	Did you attend any SHG meeting in the last six months?	Yes<br>No	1<br>0	
133	Is anyone from the household a member of self-help group?	Yes<br>No	1<br>0	
134	How frequently do you watch television?	Daily<br>Weekly<br>Bi-weekly<br>Monthly<br>Occasionally<br>Not at all<br>Don't have a TV in house	1<br>2<br>3<br>4<br>5<br>6<br>7	
135	How frequently do you listen to Radio?	Daily<br>Weekly<br>Bi-weekly<br>Monthly<br>Occasionally<br>Not at all<br>Don't have radio	1<br>2<br>3<br>4<br>5<br>6<br>7	
136	How frequently do you read newspaper or magazine?	Daily<br>Weekly<br>Bi-weekly<br>Monthly<br>Occasionally<br>Not at all<br>Don't have access to newspaper/magazine	1<br>2<br>3<br>4<br>5<br>6<br>7	
137	In the last 12 months, have you experienced following symptoms?		Yes	No
		a) Continuous coughing that lasts three or more weeks (TB)	1	0
		b) Frequent coughing	1	0
		c) Pain in the chest (TB)	1	0
		d) Coughing up blood or sputum (TB)	1	0
		e) Sweating at night (TB)	1	0
		f) Shortness of breath (Asthma)	1	0
		g) Wheezing sound while breathing (Asthma)	1	0
		h) Production of mucus (sputum) (Asthma/TB)	1	0
		i) Unexplainable weight loss (TB)	1	0
		j) Troubles while sleeping due to breathing	1	0
		k) Irritation in eyes	1	0
	CHECK: Skip to Q139a if 0 is coded in Q137 a - k			

#	Question	Answers	Codes	Skip
138	During which months do you experience these issues the most?<br>**Multiple response possible.**	January<br>February<br>March<br>April<br>May<br>June<br>July<br>August<br>September<br>October<br>November<br>December	A<br>B<br>C<br>D<br>E<br>F<br>G<br>H<br>I<br>J<br>K<br>L	
139	In the last 12 months, have you experienced following symptoms?		Yes	No
		a.) Chest pain	1	0
		b.) Nose bleed	1	0
		c.) Blood in urine	1	0
		d.) Flushing	1	0

Block 2: Marriage, Cohabitation, Husband's Occupation and migration history ,

#	Question	Answers	Codes	Skip
201	How old is your husband?	Age in completed years<br>Don't know	___<br>98	
202	How old were you when you got married?<br>?	Age in completed years	___	
203	How old were you when you started living with your husband?	Age in completed years	____	
203A	Since how many months you have been living with your husband?	In months	____	
204	Can your husband read and/or write?	No<br>Yes, can read only<br>Yes, can write only<br>Yes, can both read and write,	0<br>1<br>2<br>3	
205	What is the highest standard of formal education he completed?	If less than 11, actual class<br>Higher secondary<br>Graduate<br>Diploma/ ITI/BTech or any other vocational studies<br>Masters/Post-graduate/Doctorate<br>Non-formal education	__<br>11<br>12<br>13<br><br>14<br>96	
206	What is your husband's primary nature of job?	Not working<br>Agriculture work<br>Daily Wage (non-agricultural)<br>Diamond work<br>Garment work<br>Petty Business<br>Construction worker<br>Works in brick kiln<br>Tailoring<br>Salaried Private/Government<br>Transport sector<br>Other (Specify)	11<br>12<br><br>13<br>14<br>15<br>16<br>17<br>18<br>19<br>20<br><br>21<br>97	►230

<table>
<tr><th>#</th><th>Question</th><th>Answers</th><th>Codes</th><th>Skip</th></tr>
<tr><td>207</td><td>Where does your husband currently work?</td><td>In the village
Another village/town in this district
Outside this district but within state (specify district)
Outside state (Specify: State)</td><td>1
2
3

4</td><td>►230
►230</td></tr>
<tr><td>208</td><td>When was the first time he moved out from this district for job/work? ?</td><td>No. of years back
Before marriage, I don't know the actual time,</td><td>____
98</td><td></td></tr>
<tr><td>209</td><td>How many places has your husband visited in last five years (incase the woman has been married for less than 5 years, record the number of places husband has visited since they got married)?
?

Use probing for migration: Ask for the previous place to the current place, before that, before that, before that</td><td colspan="3">Number of places
<table>
<tr><th>Name of places</th><th>District</th><th>State</th><th>Duration of stay (in months)</th></tr>
<tr><td>Place 1</td><td></td><td></td><td></td></tr>
<tr><td>Place 2</td><td></td><td></td><td></td></tr>
<tr><td>Place 3</td><td></td><td></td><td></td></tr>
<tr><td>Place 4</td><td></td><td></td><td></td></tr>
<tr><td>Place 5</td><td></td><td></td><td></td></tr>
<tr><td>Place 6</td><td></td><td></td><td></td></tr>
<tr><td>Place 7</td><td></td><td></td><td></td></tr>
<tr><td>Place 8</td><td></td><td></td><td></td></tr>
</table></td></tr>
<tr><td>210</td><td>How frequently your husband returns to the village?</td><td>Yearly
Bi-annually
Quarterly
Monthly
Weekly
Once a while
No specific routine</td><td>1
2
3
4
5
6
7</td><td></td></tr>
<tr><td>211</td><td>Is there a particular season/month, when your husband come to house? If Yes, which are those occasions?</td><td>No sepcific season/time
Chaiti Chhath
Kartikeya Chhath (Main chhath)
Dusshera
Diwali
Saraswati Puja
Holi
Other local events (specify)
Paddy cultivation time
Taadi drinking season
Family functions (specify)
Eid
Moharram
Other (Specify)</td><td>A
B
C
D
E
F
G
H
I
J
K
L
M
Y</td><td></td></tr>
<tr><td>212</td><td>What are the reasons for your husband's visit?

Multiple responses possible.</td><td>Family function
To drink tadi
Birth of a child
Events at home
Break from work
Husband was unwell
Agricultural work
Festival
Other (Specify)</td><td>A
B
C
D
E
F
G
H
Y</td><td></td></tr>
<tr><td>213</td><td>In general, how many days your husband stays here when he comes to house?</td><td>Number of days</td><td>____</td><td></td></tr>
<tr><td>214</td><td>In general, how many days in advance do you get to know that your husband is visiting home?</td><td>Number of days</td><td>____</td><td></td></tr>
</table>

#	Question	Answers	Codes	Skip
215	In the last 12 months, which are the months when your husband came home?<br>Instruction: Record names of all the months during which woman's husband visited home in the last 12 months.	January<br>February<br>March<br>April<br>May<br>June<br>July<br>August<br>September<br>October<br>November<br>December<br>Didn't visit home in last 12 months	A<br>B<br>C<br>D<br>E<br>F<br>G<br>H<br>I<br>J<br>K<br>L<br>Y	
216	How many days ago did your husband visit home last /current time?	Number of days	____	
217	The last time/ this time husband came here, how many days in advance did you get to know that your husband is visiting home?	Number of days	____	
218	What was the primary reason of his last visit to home?	Break from work<br>Function in the house/community<br>Health issues of husband<br>Health issues of wife<br>Health issues of other family member<br>Farming<br>Meeting friends/relatives<br>Other (Specify)	11<br>12<br>13<br>14<br>15<br>16<br>17<br>97	
219	Is your husband still here?	Yes<br>No	1<br>0	►223
220	How many days did he stay during his last visit?	Number of days	____	
221	Which month he will be coming back home the next time?	January<br>February<br>March<br>April<br>May<br>June<br>July<br>August<br>September<br>October<br>November<br>December<br>Undecided	11<br>12<br>13<br>14<br>15<br>16<br>17<br>18<br>19<br>20<br>21<br>22<br>98	224
222	If undecided, which is the most likely month that he may visit?	January<br>February<br>March<br>April<br>May<br>June<br>July<br>August<br>September<br>October<br>November<br>December	11<br>12<br>13<br>14<br>15<br>16<br>17<br>18<br>19<br>20<br>21<br>22	224

<table>
<tr><th>#</th><th>Question</th><th colspan="2">Answers</th><th>Codes</th><th>Skip</th></tr>
<tr><td>223</td><td>How many more days your husband is planning to stay here before returning to his workplace?
?</td><td colspan="2">Number of days
Not planned yet
Not going again
Other (Specify)</td><td>__
88
89
97</td><td></td></tr>
<tr><td>224</td><td>How frequently your husband communicates with you when he is away?</td><td colspan="2">Everyday
2-3 times in a week
Weekly once
2-3 times in a month
Once a month
Occasionally</td><td>1
2
3
4
5
6</td><td></td></tr>
<tr><td>225</td><td>What is the primary mode of communication you and your husband use to communicate with each other?</td><td colspan="2">Own mobile phone
Mobile phone owned by another HH member
Mobile phone from neighbour
Letter
Other (Specify) ________</td><td>1
2

3
4
7</td><td></td></tr>
<tr><td>226</td><td>Have you ever visited his places of work outside district? If yes, how many times in last two years?</td><td colspan="2">Never
Number of times</td><td>0
__</td><td></td></tr>
<tr><td>227</td><td>Does your husband send remittances to you when he is away from home?</td><td colspan="2">Yes
No</td><td>1
0</td><td>
►301</td></tr>
<tr><td>228</td><td>How frequently do you receive remittances?</td><td colspan="2">Monthly
Quarterly
Bi-annually
Annually
No fixed schedule
Other (Specify)</td><td>1
2
3
4
5
7</td><td></td></tr>
<tr><td>229</td><td>How does your husband send remittances to you?</td><td colspan="2">Through bank account
Money order
Through his friends
Other (Specify)</td><td>1
2
3
7</td><td>►301
►301
►301
►301</td></tr>
<tr><td>230</td><td>Did your husband ever go outside this district for job/work?</td><td colspan="2">Yes
No</td><td>1
0</td><td>►301</td></tr>
<tr><td>231</td><td>When was the first time he moved out from this district for job/work?</td><td colspan="2">No. of years back
Before marriage, I don’t know the actual time</td><td>____
98</td><td></td></tr>
<tr><td rowspan="6">232</td><td rowspan="6">Which are the places where your husband went for job/work?
(RECORD ONLY LAST FIVE PLACES)</td><td>Name of the place</td><td>District</td><td>State</td><td rowspan="6"></td></tr>
<tr><td>Place 1</td><td></td><td></td></tr>
<tr><td>Place 2</td><td></td><td></td></tr>
<tr><td>Place 3</td><td></td><td></td></tr>
<tr><td>Place 4</td><td></td><td></td></tr>
<tr><td>Place 5</td><td></td><td></td></tr>
<tr><td>233</td><td>How frequently your husband returned to the village when he was staying away for job/work?</td><td colspan="2">Yearly
Bi-annually
Quarterly
Monthly
Weekly
Once a while
No specific routine</td><td>1
2
3
4
5
6
7</td><td></td></tr>
</table>

#	Question	Answers	Codes	Skip
234	Was there a particular season/month, when your husband used to return to house? If Yes, which were those occasion?	No sepcific season/time<br>Chaiti Chhath<br>Kartikeya Chhath (Main chhath)<br>Dusshera<br>Diwali<br>Saraswati Puja<br>Holi<br>Other local events (specify)<br>Paddy cultivation time<br>Taadi drinking season<br>Family functions (specify)<br>Eid<br>Moharram<br>Other (Specify)	A<br>B<br>C<br>D<br>E<br>F<br>G<br>H<br>I<br>J<br>K<br>L<br>M<br>Y	
235	Which were the months during which your husband used to come home most?	January<br>February<br>March<br>April<br>May<br>June<br>July<br>August<br>September<br>October<br>November<br>December	A<br>B<br>C<br>D<br>E<br>F<br>G<br>H<br>I<br>J<br>K<br>L	

Block-3: Reproductive history

#	Question	Answers	Codes	Skip
Now I would like to ask about all the pregnancies and births you have had during your life.				
301	Have you ever been pregnant?<br>?	Yes<br>No	1<br>0	 ►323
302	Are you pregnant now?	Yes<br>Unsure/no response<br>No	1<br>2	
303	[IF CURRENTLY PREGNANT]<br>Beside the current pregnancy, how many times have you been pregnant in your lifetime?<br>[IF CURRENTLY NOT PREGNANT]<br>How many times have you been pregnant in your lifetime?	 Number		
	CHECK Q302 AND Q303: Currently pregnant and first pregnancy (Q302 = 1 & Q303 = 0)	Yes<br>No	1<br>0	►319
304	How many living sons and daughters do you have?	a. Number of sons<br>b. Number of daughters<br>c. Total no. of children		
	CHECK Q304C: IF NUMBER OF CHILDREN > 1	Yes<br>No	1<br>0	 ►306
305	What is the difference in months between the age of youngest child and the child before him/her?	Number of months	—	
306	How many times have you been pregnant in last five years?	Number	——	

#	Question	Answers	Codes	Skip
307	In the last 5 years, how many children were born alive but died after birth?	Number of children died<br>None	____<br>0	
308	In the last 5 years, how many still births you had?	Number of still births<br>None	____<br>0	
309	In the last 5 years, did you have any miscarriage? If yes, how many?	Number	____	
310	In the last 5 years, did you take any medicine or visit a health facility to terminate a pregnancy after conception? If yes, how many?	Number<br>None	 0	
311	In the last 5 years, did you do anything else beside the medication to terminate a pregnancy after conception? If yes, how many?	Number<br>None	 0	
	CHECK Q310 & Q311: Abortion > 0	Yes<br>No	1<br>0	 ►31
312	How many months ago did you terminate the last pregnancy?	Number of months		
313	How many weeks pregnant were you when you terminated the last pregnancy?	Number of weeks		
314	How many children you had when you had the last abortion?	Number of children		
315	What was the primary reason you went for abortion last time?	Husband did not want a child<br>I didn't want a child<br>Unplanned pregnancy<br>I faced health complications<br>Fetus was underdeveloped<br>Female fetus<br>Male fetus<br>There was no one at home to support<br>Economic reason<br>Last child too young<br>Other (Specify) __________	11<br>12<br>13<br>14<br>15<br>16<br>17<br>18<br>19<br>20<br>97	
316	After the last abortion, did any one beside your family members, talked to you on using any family planning method?	Yes<br>No	1<br>0	
317	Did you use any family planning method after the last abortion?	Yes<br>No	1<br>0	
	CHECK Q302: CURRENTLY PREGNANT	Yes<br>No	1<br>0	►321
318	Have you ever undergone hysterectomy?	Yes<br>No	1<br>0	 ►322
319	Why did you go for hysterectomy?	Did not want any more children<br>Sterilization failed<br>Had complications after sterilization<br>Wanted a permanent solution<br>Other (Specify)	A<br>B<br>C<br>D<br>Y	
320	What was your age when you went for hysterectomy?	Age in years	—	►322
321	At the time you became pregnant currently, did you want to become pregnant then, did you want to wait until a later period, or did you not want to have any /any more children at all?,?	Then<br>Later<br>Not at all	1<br>2<br>3	►323<br>►323<br>►323
322	[ASK IF MORE THAN ONE PREGNANCY] During your last pregnancy, did you want to become pregnant then or did you want to wait until later or did you do not want to have any (more) children at all?	Wanted to become pregnant<br>Wanted to wait till a later time<br>Did not want any (more) children	1<br>2<br>3	

#	Question	Answers	Codes	Skip
323	[IF NEVER PREGNANT]<br>Would you like to have a child, or would you prefer not to have any children?<br>[IF EVER PREGNANT]<br>Would you like to have another child, or would you prefer not to have any more children?	Have (a/another) child<br>No more/none<br>Can't get pregnant<br>Undecided/don't know	1<br>2<br>3<br>4	►327<br><br>►401
324	Are you doing something to make sure you don't get pregnant anymore?	Yes<br>No	1<br>0	 ►327
325	If yes, what are you doing?	Abstinence<br>Female sterilization<br>Using other modern contraceptive methods (Specify)<br>Using traditional contraceptive methods (Specify)<br>Aayurvedic medicine<br>Hysterectomy<br>Other (Specify)	11<br>12<br>13<br>14<br>15<br>16<br>97	
326	Who suggested you about this practice?	ASHA/ANM<br>Husband<br>Relative/friend/neighbour<br>Community Mobilizer of SHG<br>Other (Specify)	1<br>2<br>3<br>4<br>7	►401
327	How long would you like to wait from now before the birth of (a/another) child?	Months<br>Soon/now<br>Can't get pregnant<br>Do not want any (more) children<br>Don't know	___<br>93<br>94<br>97<br>98	
	Check: Want to wait for more than 23 months	Yes<br>No	1<br>0	 ►401
328	Are you doing something to make sure you don't get pregnant in next two years?	Yes<br>No	1<br>0	 ►401
329	If yes, what are you doing?	Abstinence<br>Using other modern contraceptive methods<br>Using traditional contraceptive methods<br>Aayurvedic medicine<br>Not doing anything<br>Other (Specify)	1<br>2<br>3<br>4<br>5<br>7	
330	Who suggested you about this practice?	ASHA/ANM<br>Husband<br>Relative/friend/neighbour<br>Community Mobilizer of SHG<br>Other (Specify)	1<br>2<br>3<br>4<br>7	

Block 4: Use of contraceptive

#	Question	Answers	Codes	Skip
401	Which are the family planning methods you are aware of?	A. Female Sterilization<br>B. Male Sterilization<br>C. IUCD/LOOP/Copper-T<br>D. Injectables/Antara injection<br>E. Pills (Mala – D/ Chhaya)<br>F. Emergency contraception<br>G. Male condom/Nirodh<br>H. Female condom<br>I. Standard days method<br>J. Lactational amenorrhoea method<br>K. Other modern method<br>L. Rhythm method<br>M. Withdrawal<br>N. Other traditional method<br>O. Aayurvedic medicine<br>P. Not aware of any FP method<br>Q. Other (Specify)	A<br>B<br>C<br>D<br>E<br>F<br>G<br>H<br>I<br>J<br>K<br>L<br>M<br>N<br>O<br>P<br>Y	
402	Have you ever done anything to delay or avoid getting pregnant?	Yes<br>No	1<br>0	 ►501
403	Which are the family planning methods you have **<u>ever used</u>** to delay or avoid getting pregnant?	A. Female Sterilization<br>B. Male Sterilization<br>C. IUCD/LOOP/Copper-T<br>D. Injectables/Antara injection<br>E. Pills (Mala – D/ Chhaya)<br>F. Emergency contraception<br>G. Male condom/Nirodh<br>H. Female condom<br>I. Standard days method<br>J. Lactational amenorrhoea method<br>K. Other modern method<br>L. Rhythm method<br>M. Withdrawal<br>N. Other traditional method<br>O. Aayurvedic medicine<br>P. Other (Specify)	A<br>B<br>C<br>D<br>E<br>F<br>G<br>H<br>I<br>J<br>K<br>L<br>M<br>N<br>O<br>Y	
M1	CHECK Q207, Q219, Q223, Q228:<br>Husband's migration status	Currently migrant and away (Q207 > 2 & Q219 = 0)<br>Currently migrant and visiting (Q207 > 2 & Q219 = 1 & Q223 != 89)<br>Permanently returnee migrant (Q230 == 1 OR (Q207 > 2 & Q223 = 89))<br>Non-migrant (Q207 < 3 & Q230 = 0)	1<br><br>2<br><br>3<br><br>4	
404	[ASK ONLY FOR ACTIVE MIGRANTS: M1=1]<br>Did you have sexual intercourse with your husband when he visited the last time?<br>[ASK ONLY FOR VISITING MIGRANTS: M1=2]<br>Did you have sexual intercourse with your husband during his current visit?<br>[ASK ONLY FOR VISITING and NON-MIGRANTS: M1>2]<br>Did you have sexual intercourse with your husband in last 30 days?	Yes<br>No	1<br>0	

#	Question	Answers	Codes	Skip
401	Are you (or your husband) currently doing something or using any method to delay or avoid getting pregnant?	Yes<br>No	1<br>0	 ►501
402	**[ASK ONLY FOR NON-MIGRANTS, RETURNEE MIGRANTS AND VISITING MIGRANTS: M1>1]**<br>Which method(s) are you currently using?<br><br>**[ASK ONLY FOR ACTIVE MIGRANTS: M1=1]**<br>Which method(s) did you use?	A. Female Sterilization<br>B. Male Sterilization<br>C. IUCD/LOOP/Copper-T<br>D. Injectables/Antara injection<br>E. Pills (Mala – D/ Chhaya)<br>F. Emergency contraception<br>G. Male condom/Nirodh<br>H. Female condom<br>I. Standard days method<br>J. Lactational amenorrhoea method<br>K. Other modern method<br>L. Rhythm method<br>M. Withdrawal<br>N. Other traditional method<br>O. Aayurvedic medicine<br>P. Other (Specify)	A<br>B<br>C<br>D<br>E<br>F<br>G<br>H<br>I<br>J<br>K<br>L<br>M<br>N<br>O<br>Y	
	CHECK Q406: If reported more than one method besides sterilization?	Yes<br>No	1<br>0	 ►408
403	Which of these methods is/was most frequently used?	IUCD/LOOP/Copper-T<br>Injectables/Antara injection<br>Pills (Mala – D/ Chhaya)<br>Emergency contraception<br>Male condom/Nirodh<br>Female condom<br>Standard days method<br>Lactational amenorrhoea method<br>Other modern method<br>Rhythm method<br>Withdrawal<br>Other traditional method<br>Aayurvedic medicine<br>Other (Specify)	11<br>12<br>13<br>14<br>15<br>16<br>17<br>18<br>19<br>20<br>21<br>22<br>23<br>97	
404	What is your most preferred method?	Female sterilization<br>Male sterilization<br>IUCD/LOOP/Copper-T<br>Injectables/Antara injection<br>Pills (Mala – D/ Chhaya)<br>Emergency contraception<br>Male condom/Nirodh<br>Female condom<br>Standard days method<br>Lactational amenorrhoea method<br>Other modern method<br>Rhythm method<br>Withdrawal<br>Other traditional method<br>Aayurvedic medicine<br>Other (Specify)	11<br>12<br>13<br>14<br>15<br>16<br>17<br>18<br>19<br>20<br>21<br>22<br>23<br>24<br>25<br>97	

#	Question	Answers	Codes	Skip
405	Why do you prefer to use ____ the most?	A. Husband feels comfortable<br>B. Freely available<br>C. ASHA/ANM recommended<br>D. Fear of no side effects<br>E. Friend/relative recommended<br>F. Associated incentive<br>G. Provider recommended<br>H. Easily available<br>I. Provides protection for longer time<br>J. Easy to manage<br>K. Can use it without informing husband<br>L. Other (Specify)	A<br>B<br>C<br>D<br>E<br>F<br>G<br>H<br>I<br>J<br>K<br><br>Y	
	CHECK Q406: Using female sterilization (A) 406: A)	Yes<br>No	1<br>0	 ►418
406	What was your age when you went for sterilization?	Age	__	
407	Why did you go for sterilization?	A. Family size completed<br>B. Permanent solution<br>C. Good incentive amount<br>D. Husband asked me to do so<br>E. ASHA persuaded<br>F. Relative/Friends recommended<br>G. Was not keeping well<br>H. Other FP methods were not effective<br>I. Had bad experince with earlier method<br>J. Other (Specify) ________	A<br>B<br>C<br>D<br>E<br>F<br>G<br>H<br><br>I<br><br>Y	
408	Did you and your husband consulted with each other before deciding on sterilization?	Yes<br>No	1<br>0	
409	Where did you go for sterilization?	PHC/ CHC<br>Private clinic/hospital<br>District hospital/Medical college<br>Sub-divisional hospital<br>Camps<br>Other (Specify)	1<br>2<br>3<br>4<br>5<br>7	
410	In which month did you undergo sterilization?	January<br>February<br>March<br>April<br>May<br>June<br>July<br>August<br>September<br>October<br>November<br>December	10<br>11<br>12<br>13<br>14<br>15<br>16<br>17<br>18<br>19<br>20<br>21	
411	After how many months of last pregnancy, you went for sterilization?	Number of months	__	
412	Were you told about other FP methods before going for sterilization?	Yes<br>No	1<br>0	
413	Did you receive any incentive for sterilization?	Yes<br>No	1<br>0	

#	Question	Answers	Codes	Skip
	CHECK Q406: Using IUCD (C)<br>C)	Yes<br>No	1<br>0	 ►433
414	[ASK ONLY FOR ACTIVE MIGRANTS: M1=1]<br>The IUCD that you were using when your husband was here last time, is it still in place?	Yes<br>No	1<br>0	►421
415	[ASK ONLY FOR ACTIVE MIGRANTS: M1=1]<br>For how many months, did you continuously use the last IUCD you used?	Number of months		
416	Why did you get the IUCD removed?	A. Wanted to get pregnant<br>B. Experienced side effect<br>C. Have to travel far to get the IUCD<br>D. Husband refused/forced<br>E. Advised by ASHA/Health worker<br>F. Advised by friend/neighbour/ relative<br>G. Mother-in-law suggested<br>H. Heard about other womenexperiencing problems<br>I. Planning to go for sterilization<br>J. Wanted more effective method<br>K. Husband migrated [M1]M1]<br>L. Other (Specify)	A<br>B<br>C<br>D<br>E<br>F<br>G<br>H<br>I<br>J<br>K<br>Y	422
417	Since when you are using IUCD/Copper-T?	Number of months		
418	Was the current IUCD implanted within 48 hours of last child birth?<br><br>[ASK ONLY TO THOSE WHO HAVE GIVEN AT LEAST ONE BIRTH]	Yes<br>No	1<br>0	
419	Why did you go for IUCD insertion?	A. Husband asked me to do so<br>B. ASHA persuaded<br>C. Relative/Friends recommended<br>D. No side effects<br>E. Painless<br>F. Non-surgical<br>G. Had no access to other method<br>H. Gives me freedom to take it out whenever I want to<br>I. Other (Specify) ________	A<br>B<br>C<br>D<br>E<br>F<br>G<br>H<br>Y	
420	Where did you go for IUCD implantation?	PHC/ CHC<br>Private clinic/hospital<br>District hospital/Medical college<br>Sub-divisional hospital<br>Camps<br>Other (Specify)	1<br>2<br>3<br>4<br>5<br>7	
421	Did you and your husband consulted with each other before deciding to get IUCD inserted?	Yes<br>No	1<br>0	
422	In your lifetime, how many times you have got IUCD implanted?	Number of times	__	
	CHECK Q426: IUCD inserted more than once **?**	Yes<br>No	1<br>0	 ►429
423	Last time you had got IUCD implanted, for how long you kept using it?	Number of months		

#	Question	Answers	Codes	Skip
424	Why did you get the IUCD removed the last time you were using it?	A. Wanted to get pregnant<br>B. Experienced side effect<br>C. Have to travel far to get the IUCD<br>D. Husband refused/forced<br>E. Advised by ASHA/Health worker<br>F. Advised by friend/neighbour/ relative<br>G. Mother-in-law suggested<br>H. Heard about other womenexperiencing problems<br>I. Planning to go for sterilization<br>J. Wanted more effective method<br>K. Husband migrated [M1]M1]<br>L. Other (Specify)	A<br>B<br>C<br>D<br>E<br>F<br>G<br>H<br>I<br>J<br>K<br>Y	
425	[ASK ONLY TO ACTIVE AND VISITING MIGRANT]<br>Are you planning to use any FP method when your husband comes back next time? If yes, will you continue with the same method?<br><br>[ASK ONLY TO NON-MIGRANT & RETURNEE MIGRANT]<br>Are you planning to use any FP method in next 12 months? If yes, will you continue with the same method?	Yes, same method,<br>Yes, but another method,<br>No	1<br>2<br>0	►433<br><br>►432
426	Which method do you want to switch to?	A. Female Sterilization<br>B. Male Sterilization<br>C. Injectables/Antara injection<br>D. Pills (Mala – D/ Chhaya)<br>E. Emergency contraception<br>F. Male condom/Nirodh<br>G. Female condom<br>H. Standard days method<br>I. Lactational amenorrhoea method<br>J. Other modern method<br>K. Rhythm method<br>L. Withdrawal<br>M. Other traditional method<br>N. Aayurvedic medicine<br>O. Other (Specify)	A<br>B<br>C<br>D<br>E<br>F<br>G<br>H<br>I<br>J<br>K<br>L<br>M<br>N<br>Y	
427	Why you don't want to continue using IUCD?	Wanted to get pregnant<br>Experienced side effect<br>Have to travel far to get the IUCD<br>Husband refused/forced<br>Advised by ASHA/Health worker<br>Advised by friend/neighbour/ relative<br>Mother-in-law suggested<br>Heard about other women experiencing problems<br>Planning to go for sterilization<br>Wanted more effective method<br>Other (Specify)	A<br>B<br>C<br>D<br>E<br>F<br>G<br>H<br>I<br>J<br>Y	433

#	Question	Answers	Codes	Skip
428	Why you don't want to continue use any FP method?	A. Husband lives away<br>B. Husband / partner disapproved<br>C. Wanted to become pregnant<br>D. Costs too much<br>E. Health concerns/ side effects<br>F. Inconvenient to use<br>G. Fatalistic<br>H. Menopausal/ Can't get pregnant<br>I. Planning hysterctomy<br>J. Not acceptable in society/ family<br>K. Not easily available<br>L. Other (Specify)	A<br>B<br>C<br>D<br>E<br>F<br>G<br>H<br>I<br>J<br>K<br>Y	
	CHECK Q406: Using Antara (D)	Yes<br>No	1<br>0	 ►445
429	When was the first time you received Antara injection?	Number of months ago	____	
430	Till today, how many times you have taken Antara injections?	Number of times	___	
431	When was the last time you received an Antara injection?	Number of months ago	____	
432	[ASK ONLY FOR MIGRANTS: M1=< 4]<br>When you received the Antara injection last, was your husband planning to return within 3 months?	Yes<br>No	1<br>0	
433	Why did you decide to use Antara injection?	Husband asked me to do so<br>ASHA persuaded<br>Relative/Friends recommended<br>No side effects<br>Painless<br>Non-surgical<br>Effective for longer time<br>Had no access to other methods<br>Other (Specify) ________	A<br>B<br>C<br>D<br>E<br>F<br>G<br>H<br>Y	
434	Where did you go for Antara injection the last time?	PHC/ CHC<br>Private clinic/hospital<br>District hospital/Medical college<br>Sub-divisional hospital<br>Camps<br>VHSND<br>Other (Specify)	1<br>2<br>3<br>4<br>5<br>6<br>7	
435	Did you and your husband consulted with each other before deciding to take Antara injection?	Yes<br>No	1<br>0	
	CHECK Q435: Received last injection three months ago	Yes<br>No	1<br>0	►441
436	Why have you stopped taking Antara injection?	Wanted to get pregnant<br>Experienced side effect<br>Have to travel far to get the injection<br>Husband refused/forced<br>Advised by ASHA/Health worker<br>Advised by friend/neighbour/ relative<br>Mother-in-law suggested<br>Heard about other women experiencing problems<br>Planning to go for sterilization<br>Wanted more effective method<br>Husband migrated<br>Other (Specify)	A<br>B<br>C<br>D<br>E<br>F<br>G<br>H<br>I<br>J<br>K<br>Y	

#	Question	Answers	Codes	Skip
437	[ASK ONLY TO ACTIVE AND VISITING MIGRANT]<br>Are you planning to use any FP method when your husband comes back next time? If yes, will you continue with the same method?<br><br>[ASK ONLY TO NON-MIGRANT & RETURNEE MIGRANT]<br>Are you planning to use any FP method in next 12 months? If yes, will you continue with the same method?	Yes, same method,<br>Yes, but another method,<br>No	1<br>2<br>0	►445<br><br>►444
438	Which method do you want to switch to?<br><br>[ASK ONLY FOR ACTIVE MIGRANTS: M1=1]	A. Female Sterilization<br>B. Male Sterilization<br>C. IUCD/LOOP/Copper-T<br>D. Pills (Mala – D/ Chhaya)<br>E. Emergency contraception<br>F. Male condom/Nirodh<br>G. Female condom<br>H. Standard days method<br>I. Lactational amenorrhoea method<br>J. Other modern method<br>K. Rhythm method<br>L. Withdrawal<br>M. Other traditional method<br>N. Aayurvedic medicine<br>O. Other (Specify)	A<br>B<br>C<br>D<br>E<br>F<br>G<br>H<br>I<br>J<br>K<br>L<br>M<br>N<br>Y	
439	Why you don't want to continue using Antara injection?	Wanted to get pregnant<br>Experienced side effect<br>Have to travel far to get the injection<br>Husband refused/forced<br>Advised by ASHA/Health worker<br>Advised by friend/neighbour/ relative<br>Mother-in-law suggested<br>Heard about other womenexperiencing problems<br>Planning to go for sterilization<br>Wanted more effective method<br>Husband migrated<br>Other (Specify)	A<br>B<br>C<br>D<br>E<br>F<br>G<br>H<br><br>I<br>J<br>K<br>Y	445
440	Why you don't want to continue use any FP method?	A. Husband lives away<br>B. Husband / partner disapproved<br>C. Wanted to become pregnant<br>D. Costs too much<br>E. Health concerns/ side effects<br>F. Inconvenient to use<br>G. Fatalistic<br>H. Menopausal/ Can't get pregnant<br>I. Planning hysterctomy<br>J. Not acceptable in society/ family<br>K. Not easily available<br>L. Other (Specify)	A<br>B<br>C<br>D<br>E<br>F<br>G<br>H<br>I<br>J<br>K<br>Y	
	CHECK Q406: Using pill (E)	Yes<br>No	1<br>0	 ►458
441	[ASK ONLY FOR ACTIVE MIGRANTS: M1=1]<br>Are you still consuming pills?	Yes<br>No	1<br>0	►448

#	Question	Answers	Codes	Skip
442	[ASK ONLY FOR ACTIVE MIGRANTS: M1=1]<br>For how many months, did you continuously consume pills?	Number of months		
443	Why did you stop consuming pills?	Wanted to get pregnant<br>Experienced side effect<br>Have to travel far to get the pills<br>Husband refused/forced<br>Advised by ASHA/Health worker<br>Advised by friend/neighbour/ relative<br>Mother-in-law suggested<br>Heard about other womenexperiencing problems<br>Planning to go for sterilization<br>Wanted more effective method<br>Husband migrated<br>Other (Specify)	A<br>B<br>C<br>D<br>E<br>F<br>G<br>H<br>I<br>J<br>K<br>Y	449
444	Since when you are taking pills?	Number of months ago	__	
445	What type of pills primarily do you take?	Mala-D<br>Chhaya<br>Mala-N<br>Saheli<br>Other (Specify)	1<br>2<br>3<br>4<br>7	
446	How often do you take the pills?	Daily<br>Twice a week<br>Weekly once<br>No fixed schedule<br>Only when had unprotected sex<br>Whenever husband visits home	1<br>2<br>3<br>4<br>5<br>6	
447	Why did you decide to use pills?	A. Husband asked me to do so<br>B. ASHA persuaded<br>C. Relative/Friends recommended<br>D. No side effects<br>E. Painless<br>F. Non-surgical<br>G. Easily available<br>H. Had no access to other method<br>I. Other (Specify) ________	A<br>B<br>C<br>D<br>E<br>F<br>G<br>H<br>Y	
448	From where all do you get pills?	ASHA/AWW/ANM<br>Local shop/pharmacy<br>Husband gets it<br>Friends/Neighbours<br>PHC<br>VHSND<br>Other (Specify)	A<br>B<br>C<br>D<br>E<br>F<br>Y	
449	Did you and your husband consulted with each other before you started taking pills?	Yes<br>No	1<br>0	

#	Question	Answers	Codes	Skip
450	[ASK ONLY TO ACTIVE AND VISITING MIGRANT]<br>Are you planning to use any FP method when your husband comes back next time? If yes, will you continue with the same method?<br>[ASK ONLY TO NON-MIGRANT & RETURNEE MIGRANT]<br>Are you planning to use any FP method in next 12 months? If yes, will you continue with the same method?	Yes, same method,<br>Yes, but another method,<br>No	1<br>2<br>0	►458<br><br>►457
451	Which method you want to switch to?	A. Female Sterilization<br>B. Male Sterilization<br>C. IUCD/LOOP/Copper-T<br>D. Injectables/Antara injection<br>E. Emergency contraception<br>F. Male condom/Nirodh<br>G. Female condom<br>H. Standard days method<br>I. Lactational amenorrhoea method<br>J. Other modern method<br>K. Rhythm method<br>L. Withdrawal<br>M. Other traditional method<br>N. Aayurvedic medicine<br>O. Other (Specify)	A<br>B<br>C<br>D<br>E<br>F<br>G<br>H<br>I<br>J<br>K<br>L<br>M<br>N<br>Y	
452	Why you don't want to continue taking pills?	Wanted to get pregnant<br>Experienced side effect<br>Have to travel far to get the pills<br>Husband refused/forced<br>Advised by ASHA/Health worker<br>Advised by friend/neighbour/ relative<br>Mother-in-law suggested<br>Heard about other womenexperiencing problems<br>Planning to go for sterilization<br>Wanted more effective method<br>Husband migrated<br>Other (Specify)	A<br>B<br>C<br>D<br>E<br>F<br>G<br>H<br>I<br>J<br>K<br>Y	458
453	Why you don't want to continue use any FP method?	A. Husband lives away<br>B. Husband / partner disapproved<br>C. Wanted to become pregnant<br>D. Costs too much<br>E. Health concerns/ side effects<br>F. Inconvenient to use<br>G. Fatalistic<br>H. Menopausal/ Can't get pregnant<br>I. Planning hysterctomy<br>J. Not acceptable in society/ family<br>K. Not easily available<br>L. Other (Specify)	A<br>B<br>C<br>D<br>E<br>F<br>G<br>H<br>I<br>J<br>K<br>Y	
	CHECK Q406: Using emergency contraceptive (F)	Yes<br>No	1<br>0	 ►466
454	How often do you consume emergency contraceptive pills?	Always<br>Sometimes when I forget to use other contraceptives	1<br>2	

#	Question	Answers	Codes	Skip
455	From where all do you get emergency contraceptive pills?	ASHA/AWW/ANM<br>Local shop/pharmacy<br>Husband gets it<br>Friends/Neighbours<br>PHC<br>VHSND<br>Other (Specify)	A<br>B<br>C<br>D<br>E<br>F<br>Y	
456	Why did you decide to use emergency contraceptive pills?	Husband asked me to do so<br>ASHA persuaded<br>Relative/Friends recommended<br>No side effects<br>Painless<br>Non-surgical<br>Condom failure<br>Had no access to other method<br>Other (Specify) _______	A<br>B<br>C<br>D<br>E<br>F<br>G<br>H<br>Y	
457	Did you and your husband consulted with each other before you started taking emergency contraceptive pills?	Yes<br>No	1<br>0	
458	[ASK ONLY TO ACTIVE AND VISITING MIGRANT]<br>Are you planning to use any FP method when your husband comes back next time? If yes, will you continue with the same method?<br>[ASK ONLY TO NON-MIGRANT & RETURNEE MIGRANT]<br>Are you planning to use any FP method in next 12 months? If yes, will you continue with the same method?	Yes, same method,<br>Yes, but another method,<br>No	1<br>2<br>0	►466<br><br>►465
459	Which method you want to switch to?	A. Female Sterilization<br>B. Male Sterilization<br>C. IUCD/LOOP/Copper-T<br>D. Injectables/Antara injection<br>E. Pills (Mala – D/ Chhaya)<br>F. Male condom/Nirodh<br>G. Female condom<br>H. Standard days method<br>I. Lactational amenorrhoea method<br>J. Other modern method<br>K. Rhythm method<br>L. Withdrawal<br>M. Other traditional method<br>N. Aayurvedic medicine<br>O. Other (Specify)	A<br>B<br>C<br>D<br>E<br>F<br>G<br>H<br>I<br>J<br>K<br>L<br>M<br>N<br>Y	
460	Why you don't want to continue taking EC pills?	Wanted to get pregnant<br>Experienced side effect<br>Have to travel far to get the ECpill<br>Husband refused/forced<br>Advised by ASHA/Health worker<br>Advised by friend/neighbour/ relative<br>Mother-in-law suggested<br>Heard about other women experiencing problems<br>Planning to go for sterilization<br>Wanted more effective method<br>Husband migrated [M1]M1]<br>Other (Specify)	A<br>B<br>C<br>D<br>E<br>F<br>G<br>H<br>I<br>J<br>K<br>Y	466

#	Question	Answers	Codes	Skip
461	Why you don't want to continue use any FP method?	A. Husband lives away<br>B. Husband / partner disapproved<br>C. Wanted to become pregnant<br>D. Costs too much<br>E. Health concerns/ side effects<br>F. Inconvenient to use<br>G. Fatalistic<br>H. Menopausal/ Can't get pregnant<br>I. Planning hysterctomy<br>J. Not acceptable in society/ family<br>K. Not easily available<br>L. Other (Specify)	A<br>B<br>C<br>D<br>E<br>F<br>G<br>H<br>I<br>J<br>K<br>Y	
	CHECK Q406: Using condom (G OR H)	Yes<br>No	1<br>0	 ►501
462	Did you use a condom everytime you had sex with your husband?	Yes<br>No	1<br>0	
463	From where all do you get condoms?<br><br>**?**	ASHA/AWW/ANM<br>Local shop/pharmacy<br>Husband gets it<br>Friends/Neighbours<br>PHC<br>VHSND<br>Other (Specify)	A<br>B<br>C<br>D<br>E<br>F<br>Y	
464	[ASK ONLY TO ACTIVE AND VISITING MIGRANT]<br>Are you planning to use any FP method when your husband comes back next time? If yes, will you continue with the same method?<br><br>[ASK ONLY TO NON-MIGRANT & RETURNEE MIGRANT]<br>Are you planning to use any FP method in next 12 months? If yes, will you continue with the same method?	Yes, same method,<br>Yes, but another method,<br>No	1<br>2<br>0	►501<br><br>►471
465	Which method do you want to switch to?	A. Female Sterilization<br>B. Male Sterilization<br>C. IUCD/LOOP/Copper-T<br>D. Injectables/Antara injection<br>E. Pills (Mala – D/ Chhaya)<br>F. Emergency contraception<br>G. Standard days method<br>H. Lactational amenorrhoea method<br>I. Other modern method<br>J. Rhythm method<br>K. Withdrawal<br>L. Other traditional method<br>M. Aayurvedic medicine<br>N. Other (Specify)	A<br>B<br>C<br>D<br>E<br>F<br>G<br>H<br>I<br>J<br>K<br>L<br>M<br>Y	

#	Question	Answers	Codes	Skip
466	Why you don't want to continue using condoms?	Wanted to get pregnant<br>Experienced side effect<br>Have to travel far to get the condom<br>Husband refused/forced<br>Advised by ASHA/Health worker<br>Advised by friend/neighbour/ relative<br>Mother-in-law suggested<br>Heard about other womenexperiencing problems<br>Planning to go for sterilization<br>Wanted more effective method<br>Husband migrated<br>Other (Specify)	A<br>B<br>C<br>D<br>E<br>F<br>G<br>H<br><br>I<br>J<br>K<br>Y	501
467	Why you don't want to continue use any FP method?	A. Husband lives away<br>B. Husband / partner disapproved<br>C. Wanted to become pregnant<br>D. Costs too much<br>E. Health concerns/ side effects<br>F. Inconvenient to use<br>G. Fatalistic<br>H. Menopausal/ Can't get pregnant<br>I. Planning hysterctomy<br>J. Not acceptable in society/ family<br>K. Not easily available<br>L. Other (Specify)	A<br>B<br>C<br>D<br>E<br>F<br>G<br>H<br>I<br>J<br>K<br>Y	

SECTION 5: Side-effects, Reasons for non-use and intention to use

#	Question	Answers	Codes	Skip
	CHECK Q402, Q405, Q318: USED ANY CONTRACEPTION OR HAVE UNDERGONE HYSTERECTOMY	Yes<br>No	1<br>0	►503
501	Why you have not used any method to avoid pregnancy?<br>**MULTIPLE RESPONSE**	A. Husband lives away<br>B. Husband / partner disapproved<br>C. Wantto become pregnant<br>D. Don't know where to get<br>E. Non availability of any method in the village<br>F. Costs too much<br>G. Health concerns/Fear of side effects<br>H. Lack of knowledge on how to use<br>I. Inconvenient to use<br>J. Fatalistic<br>K. Menopausal/ Can't get pregnant<br>L. Not acceptable in society/ family<br>M. Other (Specify)___	A<br>B<br>C<br>D<br>E<br><br>F<br>G<br><br>H<br>I<br>J<br>K<br>L<br>Y	
502	Did you talk to your husband to use a method to avoid pregnancy?	Yes<br>No	1	
	Check Q403: IF STERILIZED OR USING IUCD, ANATARA, PILLS, CONDOMS, ECP**?**	Yes<br>No	1<br>0	 ►506
503	Did you experience any side effects post using a contraception?	Yes<br>No	1<br>0	 ►506

#	Question	Answers	Codes	Skip
504	What are the various side effects that you experienced?	Spotting Excessive bleeding<br>Changes in menstrual cycle<br>Pain in abdomen<br>Dizziness<br>Breast tenderness<br>Nausea/vomiting<br>Weight gain<br>Mood swings<br>Acne<br>Hair loss<br>Experienced unplanned pregnancy<br>Others (Specify) ______	A<br>B<br>C<br>D<br>E<br>F<br>G<br>H<br>I<br>J<br>K<br>Y	
505	The last time you experienced any side effects due to use of contraception, what did you do?	A. Contacted ASHA/ANM<br>B. Visited PHC/govt health facility<br>C. Visited a private clinic<br>D. Stopped using that method<br>E. Switched to another method<br>F. Continued using as it was cured after days<br>G. Informed husband<br>H. Checked with other women for solution<br>I. Shared with other women<br>J. Other (Specify)	A<br>B<br>C<br>D<br>E<br>F<br>G<br>H<br>I<br>Y	
506	Have you heard about any other women experiencing any problem with use of a FP method?	Yes<br>No	1<br>0	 ►509
507	What were the kind of problems other women were experiencing?	Spotting Excessive bleeding<br>Changes in menstrual cycle<br>Pain in abdomen<br>Dizziness<br>Breast tenderness<br>Nausea/vomiting<br>Weight gain<br>Mood swings<br>Acne<br>Hair loss<br>Experienced unplanned pregnancy<br>Others (Specify) ______	A<br>B<br>C<br>D<br>E<br>F<br>G<br>H<br>I<br>J<br>K<br>Y	509
508	Which method the other women in this area are using which resulted in unwanted pregnancy?	A. Female Sterilization<br>B. Male Sterilization<br>C. IUCD/LOOP/Copper-T<br>D. Injectables/Antara injection<br>E. Pills (Mala – D/ Chhaya)<br>F. Emergency contraception<br>G. Male condom/Nirodh<br>H. Female condom<br>I. Standard days method<br>J. Lactational amenorrhoea method<br>K. Other modern method<br>L. Rhythm method<br>M. Withdrawal<br>N. Other traditional method<br>O. Aayurvedic medicine<br>P. Other (Specify)	A<br>B<br>C<br>D<br>E<br>F<br>G<br>H<br>I<br>J<br>K<br>L<br>M<br>N<br>O<br>Y	

#	Question	Answers	Codes	Skip
509	**[Ask only if at least 1 currently married women in the age group 15-49]**<br>Do other currently married women in this household are using any contraceptive currently?	Yes<br>No<br>Don't know	1<br>0<br>8	 ►511<br>►511
510	Which method the other women in this household are using?<br><br>**MULTIPLE RESPONSE**	A. Female Sterilization<br>B. Male Sterilization<br>C. IUCD/LOOP/Copper-T<br>D. Injectables/Antara injection<br>E. Pills (Mala – D/ Chhaya)<br>F. Emergency contraception<br>G. Male condom/Nirodh<br>H. Female condom<br>I. Standard days method<br>J. Lactational amenorrhoea method<br>K. Other modern method<br>L. Rhythm method<br>M. Withdrawal<br>N. Other traditional method<br>O. Aayurvedic medicine<br>P. Other (Specify)	A<br>B<br>C<br>D<br>E<br>F<br>G<br>H<br>I<br>J<br>K<br>L<br>M<br>N<br>O<br>Y	
	CHECK Q405: Currently using any contraception or undergone hysterectomy	Yes<br>No	1<br>0	►513
511	[ASK ONLY TO ACTIVE AND VISITING MIGRANT]<br>Do you think you will use a contraceptive method when your husband comes back next time?<br>[ASK ONLY TO NON-MIGRANT & RETURNEE MIGRANT]<br>Do you think you will use a contraceptive method in next 12 months?	Yes<br>No	1<br>0	
512	Which method you would like to use?	Female Sterilization<br>Male Sterilization<br>IUCD/LOOP/Copper-T<br>Injectables/Antara injection<br>Pills (Mala – D/ Chhaya)<br>Emergency contraception<br>Male condom/Nirodh<br>Female condom<br>Standard days method<br>Lactational amenorrhoea method<br>Other modern method<br>Rhythm method<br>Withdrawal<br>Other traditional method<br>Aayurvedic medicine<br>Other (Specify)	11<br>12<br>13<br>14<br>15<br>16<br>17<br>18<br>19<br>20<br>21<br>22<br>23<br>24<br>25<br>97	
512A	Are you satisfied with the contraceptive method you are currently using?	Yes<br>No	1<br>2	
513	Who mainly decides on use of a contraceptive method? Would you say it was your decision alone, your husband's decision alone, both of you decided together, provider decided, or In-laws decided or someone else?	Respondent's decided alone/<br>Husband decided alone<br>Both decided<br>Provider decided<br>In-Laws<br>Someone else decided	1<br>2<br>3<br>4<br>5<br>6	

#	Question	Answers	Codes	Skip
514	How often your use of contraceptive method is influenced by the practices followed by other women in the community/ neighborhood?	Always<br>Sometimes<br>Occasionally<br>Never	1<br>2<br>3<br>4	
515	How often your choice of contraceptive method is influenced by the practices followed by other women in the community/ neighborhood?	Always<br>Sometimes<br>Occasionally<br>Never	1<br>2<br>3<br>4	
516	How often your use of contraceptive method is influenced by the availability of a method in community/ neighborhood?	Always<br>Sometimes<br>Occasionally<br>Never	1<br>2<br>3<br>4	
517	How often your choice of contraceptive method is influenced by the availability of a method in community/ neighborhood?	Always<br>Sometimes<br>Occasionally<br>Never	1<br>2<br>3<br>4	
518	How often your use of a contraceptive method is influenced by the experience of friends/relatives in the neighborhood?	Always<br>Sometimes<br>Occasionally<br>Never	1<br>2<br>3<br>4	
519	How often your choice of a contraceptive method is influenced by the experience of friends/relatives in the neighborhood?	Always<br>Sometimes<br>Occasionally<br>Never	1<br>2<br>3<br>4	
520	How often do you discuss on various topics of family planning with friends/relatives in the neighborhood?	Always<br>Sometimes<br>Occasionally<br>Never	1<br>2<br>3<br>4	
521	With whom do you discuss about various issues and topics related to FP?	A. None<br>B. ASHA<br>C. ANM<br>D. FP Counsellor<br>E. Women with NM husband<br>F. Women with migrant husband<br>G. Mother/Sister-in-law<br>H. Mother/Sister<br>I. Other relatives<br>J. Community mobilizer/Group leader<br>K. SHG member<br>L. Husband<br>M. DoctorProvider<br>N. Other (Specify)	A<br>B<br>C<br>D<br>E<br>F<br>G<br>H<br>I<br>J<br>K<br>L<br>M<br>Y	►523
522	In general, what are the different topics (related to healthcare) you discuss with friends/relatives in the neighborhood?	A. Desired family size<br>B. About new FP methods<br>C. Availability of FP methods<br>D. Use of FP methods<br>E. Method side-effects<br>F. Traditional methods<br>G. Myths related to a method<br>H. Other (Specify) ___	A<br>B<br>C<br>D<br>E<br>F<br>G<br>Y	
523	In last 6 months, has there been any discussion on Family Planning during any of the SHG meeting?<br>[ASK ONLY TO SHG MEMBERS]	Yes<br>No	1<br>0	

Block: 6 Practices during last two births दो बच्चों के जन्म के बीच प्रमुख घटनाएं

#	Question	Answers	Codes	Skip
	CHECK: Q305 < 60 monthsand [total number of children > 1 | Q301 = 0	Yes<br>No	1<br>0	 ►701
601	How many months your husband was at home during birth of last and second last child?	Number of months<br>Always present	___<br>96	
602	Did you (or your husband) used any method or do something to delay or avoid getting pregnant between the birth of last and second last child?	Yes<br>No	1<br>0	 ►605
603	Which method(s) did you use?	A. Female Sterilization<br>B. Male Sterilization<br>C. IUCD/LOOP/Copper-T<br>D. Injectables/Antara injection<br>E. Pills (Mala – D/ Chhaya)<br>F. Emergency contraception<br>G. Male condom/Nirodh<br>H. Female condom<br>I. Standard days method<br>J. Lactational amenorrhoea method<br>K. Other modern method<br>L. Rhythm method<br>M. Withdrawal<br>N. Other traditional method<br>O. Aayurvedic medicine<br>P. Other (Specify)	A<br>B<br>C<br>D<br>E<br>F<br>G<br>H<br>I<br>J<br>K<br>L<br>M<br>N<br>O<br>Y	
	CHECK Q603: If reported more than one method besides sterilization?	Yes<br>No	1<br>0	 ►605
604	Which of these methods was most frequently used?	IUCD/LOOP/Copper-T<br>Injectables/Antara injection<br>Pills (Mala – D/ Chhaya)<br>Emergency contraception<br>Male condom/Nirodh<br>Female condom<br>Standard days method<br>Lactational amenorrhoea method<br>Other modern method<br>Rhythm method<br>Withdrawal<br>Other traditional method<br>Aayurvedic medicine<br>Other (Specify)	11<br>12<br>13<br>14<br>15<br>16<br>17<br>18<br>19<br>20<br>21<br>22<br>23<br>97	
605	[ASK IF WOMEN HAD ABORTION/ MISCARRIEGE IN LAST 5 YEARS]<br>Did you have to undergo any miscarriage OR terminate any pregnancy between the birth of last and second last child?	Yes<br>No	1<br>0	

Block: 7 Contact with Health Care Provider and Media Exposure On FP

स्वास्थ्य कार्यकर्ता से संपर्क और संचार साधनों द्वारा परिवार नियोजन के बारे में जानकारी

#	Question	Answers	Codes	Skip
701	In the last 12 months, have you met with ASHA/ANM or any other health worker?	a. ASHA<br>b. ANM<br>c. Other health worker	1<br>1<br>1	0<br>0<br>0
**If 0 is coded in 701a to 701c then skip to Q708**				
702	How often have you met with ASHA/ANM or any other health worker in last 12 months?	Almost every month<br>At least once in two months<br>At least once every 3 months<br>Once or twice in a year	1<br>2<br>3<br>4	
703	How many times ASHA/ANM or any other health worker met you in last 3 months?	No meeting<br>Number of times	0	►705
704	What are the various topics ASHA/ANM or any other health worker talked about when you met them in last 3 months?<br><br>**?**<br><br>|	A. Child health Immunization<br>B. Water and sanitation issues<br>C. Family planning<br>D. Antenatal Care<br>E. Delivery Care<br>F. Birth Preparedness<br>G. Complication readiness<br>H. Postnatal Care<br>I. Government Incentive Programs<br>J. Disease Prevention<br>K. Nutrition/Health Education<br>L. Menstrual Hygiene<br>M. Other (Specify)	A<br>B<br>C<br>D<br>E<br>F<br>G<br>H<br>I<br>J<br>K<br>L<br>Y	
705	When was the last time ASHA/ANM discussed with you on FP methods?	Never discussed<br>Number of days ago	0	►708
706	What FP specific topics did ASHA/ ANM discuss in the last 12 months?<br><br>**MULTIPLE RESPONSE POSSIBLE**	Source to procure family planning methods<br>When to use FP methods<br>Advantages and disadvantages of different methods<br>Talked only about sterilization<br>Incentives associated with adopting different methods<br>Other (Specify)	A<br>B<br>C<br>D<br>E<br>Y	
707	How much time does ASHA/ANM spend in each visit?	Time spent (in minutes)		
708	Did you attend Village Health, Sanitation and Nutrition Day (VHSND or local name) in last 3 months?	Yes<br>No	1<br>0	 ►710
709	In the last 3 VHSNDs, did anyone talk with you about family planning?	Yes<br>No	1<br>0	
710	Which of the methods are easy to access with regards to the cost associated with it?<br><br>**MULTIPLE RESPONSE POSSIBLE**	A. Female sterilization<br>B. Male sterilization<br>C. IUCD/LOOP/Copper-T<br>D. Injectables/Antara injection<br>E. Pills (Mala – D/ Chhaya)<br>F. Emergency contraception<br>G. Male condom/Nirodh<br>H. Female condom<br>I. Standard days method<br>J. Aayurvedic medicine<br>K. Other (Specify)	A<br>B<br>C<br>D<br>E<br>F<br>G<br>H<br>I<br>J<br>Y	

#	Question	Answers	Codes	Skip
711	Which of the methods are easy to access with regards to the distance traveled to get it?<br><br>**MULTIPLE RESPONSE POSSIBLE**	A. Female sterilization<br>B. Male sterilization<br>C. IUCD/LOOP/Copper-T<br>D. Injectables/Antara injection<br>E. Pills (Mala – D/ Chhaya)<br>F. Emergency contraception<br>G. Male condom/Nirodh<br>H. Female condom<br>I. Standard days method<br>J. Aayurvedic medicine<br>K. Other (Specify)	A<br>B<br>C<br>D<br>E<br>F<br>G<br>H<br>I<br>J<br>Y	
	**CHECK M1: ACTIVE OR VISTING MIGRANT (M1 < 3)**	**Yes**<br>**No**	**1**<br>0	 ►717
712	Does ASHA/ANM or any other health worker keep a track of your husband's return?	Yes<br>No	1<br>0	
713	In general, does ASHA/ANM or any other health worker visit you when your husband is away for work?	Yes<br>No	1<br>0	 ►715
714	Does ASHA/ANM or any other health worker specifically talk about FP methods in those visits?	Yes<br>No	1<br>0	
715	Did ASHA/ANM or any other health worker meet you when your husband was here last time?	Yes<br>No	1<br>0	
716	Did you receive condom, pills or emergency contraceptive from the ASHA/ANM or any other health worker when your husband was home or was about to return the last time?	Yes<br>No	1<br>0	
717	In the last 6 months have you heard about family planning on the radio?	Yes<br>No	1<br>0	
718	In the last 6 months have you seen anything about family planning on the television?	Yes<br>No	1<br>0	
719	In the last 6 months have you read about family planning in a newspaper or magazine?	Yes<br>No	1<br>0	
720	In the last 6 months have you seen anything about family planning on a wall painting or hoarding?	Yes<br>No	1<br>0	
	**STIGMA RELATED QUESTIONS**<br>**Suppose that you were planning to get services related to contraceptives and family planning next week. Please keep this scenario in mind and tell me if you: strongly disagree, disagree, agree or strongly agree with the following statements.**			
721	I would feel embarrassed about wanting more information about contraceptives and family planning services.	Strongly disagree<br>Disagree<br>Agree<br>Strongly agree	1<br>2<br>3<br>4	
722	I would be afraid of being seen by someone I knew at the facility asking for contraceptives.	Strongly disagree<br>Disagree<br>Agree<br>Strongly agree	1<br>2<br>3<br>4	
723	I would be worried about what my family /in-laws would say if they found out that I needed contraceptive or family planning services.	Strongly disagree<br>Disagree<br>Agree<br>Strongly agree	1<br>2<br>3<br>4	

#	Question	Answers	Codes	Skip
724	I would be worried about what people in my community would say about me if they found out I needed contraceptives and family planning.|	Strongly disagree<br>Disagree<br>Agree<br>Strongly agree	1<br>2<br>3<br>4	
725	I would feel embarrassed talking to ASHA/provider about contraceptives and family planning.	Strongly disagree<br>Disagree<br>Agree<br>Strongly agree	1<br>2<br>3<br>4	
726	I would be worried that my husband/family member would not support me	Strongly disagree<br>Disagree<br>Agree<br>Strongly agree	1<br>2<br>3<br>4	
727	I would want a female friend to come with me to get contraceptives and family planning.	Strongly disagree<br>Disagree<br>Agree<br>Strongly agree	1<br>2<br>3<br>4	
728	I would be worried that religious leaders in my community would not support me.	Strongly disagree<br>Disagree<br>Agree<br>Strongly agree	1<br>2<br>3<br>4	
729	I am worried that contraceptive and family planning providers will not keep my personal and health information confidential.	Strongly disagree<br>Disagree<br>Agree<br>Strongly agree	1<br>2<br>3<br>4	
730	I would be worried that the cost of the contraceptive and family planning services would be too high and are beyond my ability to buy.	Strongly disagree<br>Disagree<br>Agree<br>Strongly agree	1<br>2<br>3<br>4	

Block 8: Decision making निर्णय लेने में भूमिका

#	Question	Response	Codes	Skip
	Who mainly takes decision regarding following?			
801	Decisions regarding how the money you earn will be used?	Respondent<br>Husband<br>Respondent and Husband jointly<br>Others	1<br>2<br>3<br>4	
802	Decisions regarding how husband's earnings will be used?	Respondent<br>Husband<br>Respondent and Husband jointly<br>Others	1<br>2<br>3<br>4	
803	Decisions about health care for yourself?	Respondent<br>Husband<br>Respondent and Husband jointly<br>Others	1<br>2<br>3<br>4	
804	Decisions about family planning?	Respondent<br>Husband<br>Respondent and Husband jointly<br>Others	1<br>2<br>3<br>4	
805	Decisions about use of a contraceptive method?	Respondent<br>Husband<br>Respondent and Husband jointly<br>Others	1<br>2<br>3<br>4	

#	Question	Response	Codes	Skip
806	Decisions regarding self, like shopping, working etc.?	Respondent<br>Husband<br>Respondent and Husband jointly<br>Others	1<br>2<br>3<br>4	
807	Decisions about kids, their education etc.?	Respondent<br>Husband<br>Respondent and Husband jointly<br>Others	1<br>2<br>3<br>4	
808	Decision about household purchases such as buying household goods, food, jewelry and property?	Respondent<br>Husband<br>Respondent and Husband jointly<br>Others	1<br>2<br>3<br>4	
809	Decisions about selling and mortgaging important household assets?	Respondent<br>Husband<br>Respondent and Husband jointly<br>Others	1<br>2<br>3<br>4	
810	Decisions about your visit to family or relatives?	Respondent<br>Husband<br>Respondent and Husband jointly<br>Others	1<br>2<br>3<br>4	
811	Decision about husband’s travel?	Respondent<br>Husband<br>Respondent and Husband jointly<br>Others	1<br>2<br>3<br>4	
